# The PfAlba1 RNA-binding protein is an important regulator of translational timing in *Plasmodium falciparum* blood stages

**DOI:** 10.1186/s13059-015-0771-5

**Published:** 2015-09-28

**Authors:** Shruthi Sridhar Vembar, Cameron Ross Macpherson, Odile Sismeiro, Jean-Yves Coppée, Artur Scherf

**Affiliations:** Unité Biologie des Interactions Hôte-Parasite, Département de Parasites et Insectes Vecteurs, Institut Pasteur, Paris, 75015 France; CNRS, ERL 9195, Paris, 75015 France; INSERM, UMR 1201, Paris, 75015 France; Plate-forme 2, Transcriptome et Epigenome, Institut Pasteur, Paris, 75015 France

## Abstract

**Background:**

Transcriptome-wide ribosome occupancy studies have suggested that during the intra-erythrocytic lifecycle of *Plasmodium falciparum*, select mRNAs are post-transcriptionally regulated. A subset of these encodes parasite virulence factors required for invading host erythrocytes, and are currently being developed as vaccine candidates. However, the molecular mechanisms that govern post-transcriptional regulation are currently unknown.

**Results:**

We explore the previously identified DNA/RNA-binding protein PfAlba1, which localizes to multiple foci in the cytoplasm of *P. falciparum* trophozoites. We establish that PfAlba1 is essential for asexual proliferation, and subsequently investigate parasites overexpressing epitope-tagged PfAlba1 to identify its RNA targets and effects on mRNA homeostasis and translational regulation. Using deep sequencing of affinity-purified PfAlba1-associated RNAs, we identify 1193 transcripts that directly bind to PfAlba1 in trophozoites. For 105 such transcripts, 43 % of which are uncharacterized and 13 % of which encode erythrocyte invasion components, the steady state levels significantly change at this stage, evidencing a role for PfAlba1 in maintaining mRNA homeostasis. Additionally, we discover that binding of PfAlba1 to four erythrocyte invasion mRNAs, Rap1, RhopH3, CDPK1, and AMA1, is linked to translation repression in trophozoites whereas release of these mRNAs from a PfAlba1 complex in mature stages correlates with protein synthesis.

**Conclusions:**

We show that PfAlba1 binds to a sub-population of asexual stage mRNAs and fine-tunes the timing of translation. This mode of post-transcriptional regulation may be especially important for *P. falciparum* erythrocyte invasion components that have to be assembled into apical secretory organelles in a highly time-dependent manner towards the end of the parasite’s asexual lifecycle.

**Electronic supplementary material:**

The online version of this article (doi:10.1186/s13059-015-0771-5) contains supplementary material, which is available to authorized users.

## Background

*Plasmodium falciparum*, the causal agent of the most deadly form of malaria [1], is a protozoan parasite belonging to the phylum Apicomplexa that undergoes complex lifecycle transitions in the mosquito vector and human host. Pathology of *P. falciparum* infection manifests during the 48-hour asexual, intra-erythrocytic developmental cycle (IDC), which is composed of three morphologically and metabolically distinct stages: ring (0–24 h), trophozoite (24–38 h) and schizont (38–48 h). Merozoites produced at the end of the IDC re-invade erythrocytes and establish malaria infection. Additionally, a small percentage of parasites generated during the IDC commit to sexual development (*i.e.*, male and female gametocytes) and subsequent transmission to the mosquito vector. Notably, vaccine production against the asexual stages has not met with great success [2], and the parasite is evolving resistance to currently available anti-malarial drugs [3, 4]. Therefore, a comprehensive understanding of essential processes that control the IDC may lead to new targets for intervention strategies.

Transcriptomic studies of the *P. falciparum* IDC revealed a cyclic pattern of gene expression, with more than 50 % of transcripts attaining peak expression at only one stage of the IDC [5, 6]. Yet, little is known about its control beyond the epigenetic regulation of multigene families such as *var*, *stevor*, and *rifin* [7, 8] and the specialized regulatory roles of some of the 27 putative ApiAP2 transcription factors [9, 10]. Moreover, recent high throughput sequencing studies have shown that >80 % of the parasite genome is pervasively transcribed in a monocistronic manner during the IDC [11, 12], hinting at robust post-transcriptional regulation to fine-tune stage-specific IDC expression. Such a mode of regulation is further supported by an enrichment of genes predicted to encode RNA-binding proteins (RBPs) in the *P. falciparum* genome [13]. For example, we recently found that in ring stages the chromatin-associated exoribonuclease PfRNaseII controls the post-transcriptional silencing of nascent RNA synthesized from a subset of genes, including virulence genes encoding surface adhesion molecules linked to severe malaria [14].

Thereafter, when the *P. falciparum* transcriptome was compared with the proteome [15–17], a delay in translation was observed for a subset of mRNA molecules (~30 %) with a median delay time of 11–18 hours. This suggested that once these mRNAs have been transcribed, they are post-transcriptionally regulated to achieve ‘just-in-time’ translation [16]. Two recent reports support this hypothesis to varying degrees: Bunnik *etal*. [18] determined the polysome occupancy profiles of different stages of the IDC and found that polysome occupancy was delayed for 1280 mRNAs (31 % of the total transcriptome) compared with peak steady state mRNA levels. On the other hand, Caro *etal*. [19] used ribosome profiling to analyze ribosome-associated RNAs at nucleotide resolution and concluded that transcription and translation are tightly coupled, with 8.3 % of the IDC transcriptome apparently being translationally regulated. Important virulence factors identified in these studies include erythrocyte invasion components such as PfRON proteins, PfRhopH3, PfEBA181, PfRh3, and PfMSPs (merozoite surface proteins), and early ring stage proteins. However, our understanding of the molecular basis of translational regulation in asexual stages is still in its infancy.

We previously established that members of the archaeal DNA/RNA-binding PfAlba family [20, 21], PfAlba1–4, bind to RNA *in vitro* and that PfAlba1, 2, and 4 localize to the perinuclear region in rings and to punctate loci in the cytoplasm of trophozoites and schizonts, reminiscent of RNA storage/processing centers [20]. In this work, we explore the role of the PfAlba proteins in post-transcriptional gene regulation during the IDC. We focused on PfAlba1, a 27 kDa protein with two RNA-binding domains, an N-terminal Alba domain that is conserved amongst all PfAlba proteins and a C-terminal arginine/glycine-rich (RGG) domain. Because we were unable to knockout or knockdown PfAlba1 in asexual blood stages, we overexpressed the protein and found that this perturbed the steady state levels of a subset of transcripts in trophozoite stages, suggesting that PfAlba1 plays a role in maintaining mRNA homeostasis during the IDC. To understand this phenotype, we identified PfAlba1’s RNA interactome using high throughput sequencing and measured significant changes in the levels of over 100 PfAlba1-bound mRNAs. Moreover, for a few mRNAs encoding proteins involved in erythrocyte invasion, such as PfRhopH3 and PfAMA1, we discovered that PfAlba1 binding correlated to translation repression, with release from PfAlba1 complexes coinciding with efficient translation. To our knowledge, our results demonstrate for the first time an important role for PfAlba1 in post-transcriptional fine-tuning of translation during asexual blood stages of *P. falciparum*.

## Results

### PfAlba1 is essential, but can be overexpressed from an episome with a C-terminal Ty1 tag in *P. falciparum* blood stages

**Fig. 1:**
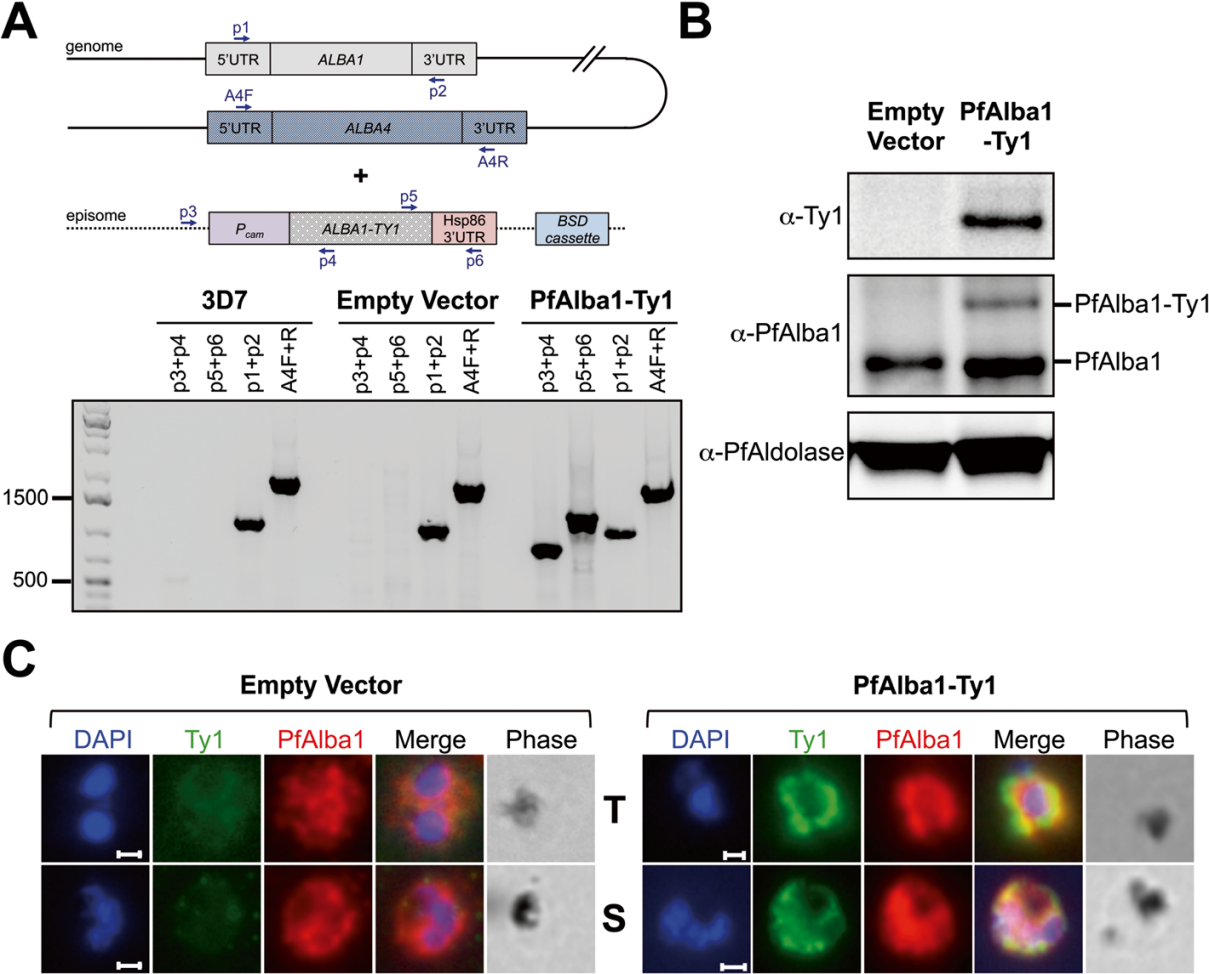
C-terminally tagged PfAlba1-Ty1 was successfully expressed from an episome in *P. falciparum* asexual blood stages. **a** The transfection and maintenance of the pPfAlba1-Ty1C plasmid was confirmed by PCR of genomic DNA. Primer pairs targeted endogenous *ALBA1* (*p1+p2*; see Additional file 11 for primer sequences), the *ALBA1-TY1* locus on the episome (*p3+p4* and *p5+p6*), and the unrelated *ALBA4* genomic locus (*A4F+R*), in either untransfected 3D7 parasites or parasites harboring the empty vector or pPfAlba1-Ty1C. The scheme (*top panel*) is not drawn to scale; *UTR =* untranslated region. The leftmost lane (*bottom panel*) represents the size marker, GeneRuler 1 kb Plus DNA Ladder (Fermentas, Life Technologies). **b** PfAlba1-Ty1 was detected in protein lysates prepared from empty vector or PfAlba1-Ty1 parasites by denaturing gel electrophoresis and western blotting with mouse anti-Ty1 antibodies or anti-PfAlba1 antibodies. PfAldolase served as a loading control. **c** Immunofluorescence assays were used to determine the localization of PfAlba1-Ty1 in trophozoite (*T*) and schizont (*S*) stages of PfAlba1-Ty1 parasites. Empty vector transfectants served as a negative control. Antibodies used included mouse anti-Ty1 (*green*) or anti-PfAlba1 (*red*). Nuclei were labeled with DAPI (*blue*). Scale bar represents 1 μM

Numerous attempts to either knock out the *ALBA1* (PF3D7_0803200) locus or generate inducible protein knockdown parasites for PfAlba1 were unsuccessful (Figure S1 in Additional file 1). After three independent transfections with two different plasmid constructs (Figure S1a in Additional file 1), we did not obtain gene knockout parasites, indicating that disruption of the *ALBA1* locus was deleterious to *P. falciparum* intra-erythrocytic growth. Thereafter, we adopted a conditional protein knockdown approach, wherein we modified the *ALBA1* locus to express PfAlba1 tagged with the *Escherichia coli* DHFR destabilization domain (eDHFR), which is stabilized by the small molecule trimethroprin (TMP) [22] (Figure S1b in Additional file 1). When ALBA1-eDHFR-HA transgenic parasites were grown in the absence of TMP, we did not observe a significant reduction in the levels of PfAlba1-eDHFR-HA, even after 10 days (Figure S1c in Additional file 1, day 7 and day 10 panels), indicating that the tagged protein is refractory to the stabilizing drug. Finally, we generated parasites hypermorphic for *ALBA1*, by transfecting 3D7 with a plasmid encoding a C-terminally tagged version of PfAlba1, PfAlba1-Ty1, from the heterologous calmodulin promoter (*P*_*cam*_) (Fig. 1a). Using western blotting, we detected PfAlba1-Ty1 with anti-Ty1 and anti-PfAlba1 antibodies (Fig. 1b) and when we analyzed the localization of the tagged protein in trophozoite and schizont stages using immunofluorescence assays, we found that PfAlba1-Ty1 was predominantly cytoplasmic (Fig. 1c) as was described for native PfAlba1 [20].

### Excess levels of PfAlba1 inhibit intra-erythrocytic growth of *P. falciparum*

**Fig. 2:**
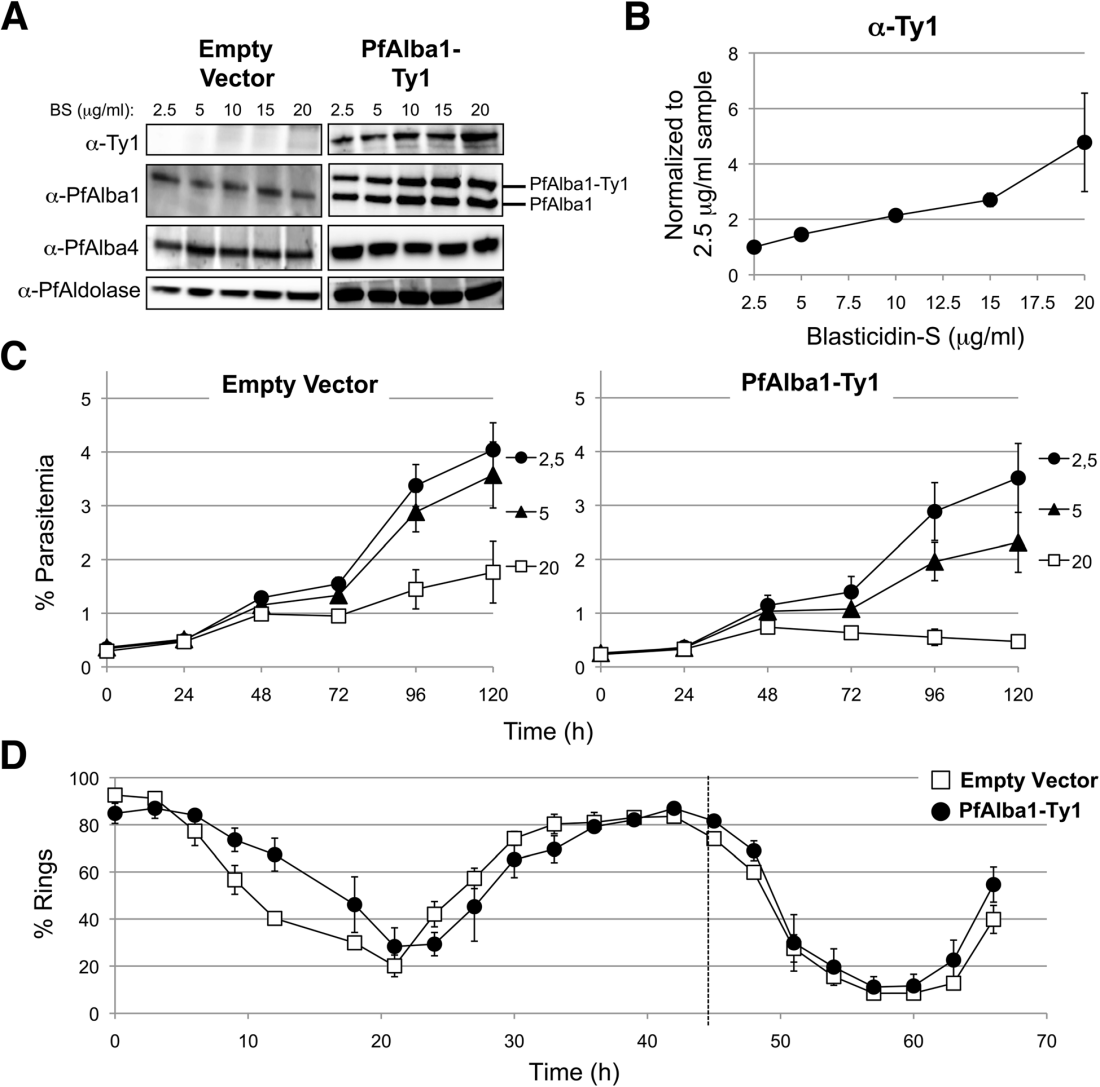
PfAlba1 overexpression reduces intra-erythrocytic growth of *P. falciparum*. **a** Protein lysates prepared from empty vector or PfAlba1-Ty1 transfectants grown in the presence of increasing concentrations of blasticidin-S (*BS*; 2.5–20 μg/ml) were separated by denaturing gel electrophoresis and analyzed by western blotting with mouse anti-Ty1 antibodies, anti-PfAlba1 antibodies, or anti-PfAlba4 antibodies. PfAldolase served as a loading control. **b** The levels of PfAlba1-Ty1, as detected by the anti-Ty1 antibodies in (**a**), were measured by densitometry. For each BS concentration, after normalizing the PfAlba1-Ty1 signal to the PfAldolase signal, data were normalized to the 2.5 μg/ml sample. Data represent the means of a minimum of three independent experiments ± standard error (S.E.; *error bars*). **c** The growth of empty vector (*left panel*) or PfAlba1-Ty1 (*right panel*) parasites was measured by flow cytometry for 5 days in the presence of the indicated concentrations of BS (in μg/ml). The *y-axis* denotes the percentage parasitemia at each time point. Data represent the means of a minimum of three independent experiments ± S.E. (*error bars*). **d** The ~48 h IDC of empty vector or PfAlba1-Ty1 transfectants was monitored by flow cytometry. The *y-axis* denotes the percentage of ring stages at each time point. The *vertical dashed line* represents one replication cycle. Data represent the means of three independent experiments ± S.E. (*error bars*)

Before functionally characterizing PfAlba1-Ty1, we explored if we could modulate the expression levels of the tagged protein during the *P. falciparum* IDC, and if so, whether the parasite had a tolerance threshold for PfAlba1-Ty1 levels. When we treated 3D7+PfAlba1-Ty1 parasites that were initially growing with 2.5 μg/ml blasticidin-S (BS) in the growth medium with increasing amounts of BS for two days, PfAlba1-Ty1 protein levels gradually increased (Fig. 2a), reaching a maximum of fivefold upregulation at 20 μg/ml BS relative to 2.5 μg/ml BS (Fig. 2b). We also observed that the levels of endogenous PfAlba1 increased in a BS dose-dependent manner (Fig. 2a; Figure S2a in Additional file 1), but not of a second Alba family protein PfAlba4 (Fig. 2a) and two other orthologs, PfAlba2 and PfAlba3 (data not shown), indicating that overexpression of PfAlba1-Ty1 directly affected endogenous PfAlba1 levels. Note that, even at 2.5 μg/ml BS, PfAlba1-Ty1 transfectants express higher levels of total PfAlba1 compared with the empty vector control (Figs. 1b and 2a).

We subsequently followed the growth of synchronized 3D7+PfAlba1-Ty1 ring stage parasites treated with different concentrations of BS using flow cytometry and found that, after two replication cycles, there was a marked reduction in the parasitemia of 3D7+PfAlba1-Ty1 parasites at 10, 15, and 20 μg/ml BS compared with 3D7+empty vector (Fig. 2c; Figure S2b in Additional file 1). Even at 5 μg/ml BS, PfAlba1-Ty1 transfectants showed a slightly lower multiplication rate compared with empty vector transfectants (Fig. 2c), indicating that a modest 1.5-fold increase in PfAlba1-Ty1 levels was sufficient to delay parasite growth. We used this concentration of BS for further analysis and measured the cell cycle time of 3D7+PfAlba1-Ty1 parasites by flow cytometry. However, we did not observe a significant difference in the progression of ring to late stages between 3D7+empty vector and 3D7+PfAlba1-Ty1 parasites (Fig. 2d), implying that growth reduction of PfAlba1-Ty1 transfectants was not due a change in cell cycle time. Overall, our results show that excess amounts of PfAlba1 reduce *P. falciparum* intra-erythrocytic growth in a dose-dependent manner, but not by altering the onset of DNA replication and schizogony.

### Immunoprecipitation analysis identifies 1665 transcripts that associate with PfAlba1-Ty1 complexes in trophozoite stages

**Fig. 3:**
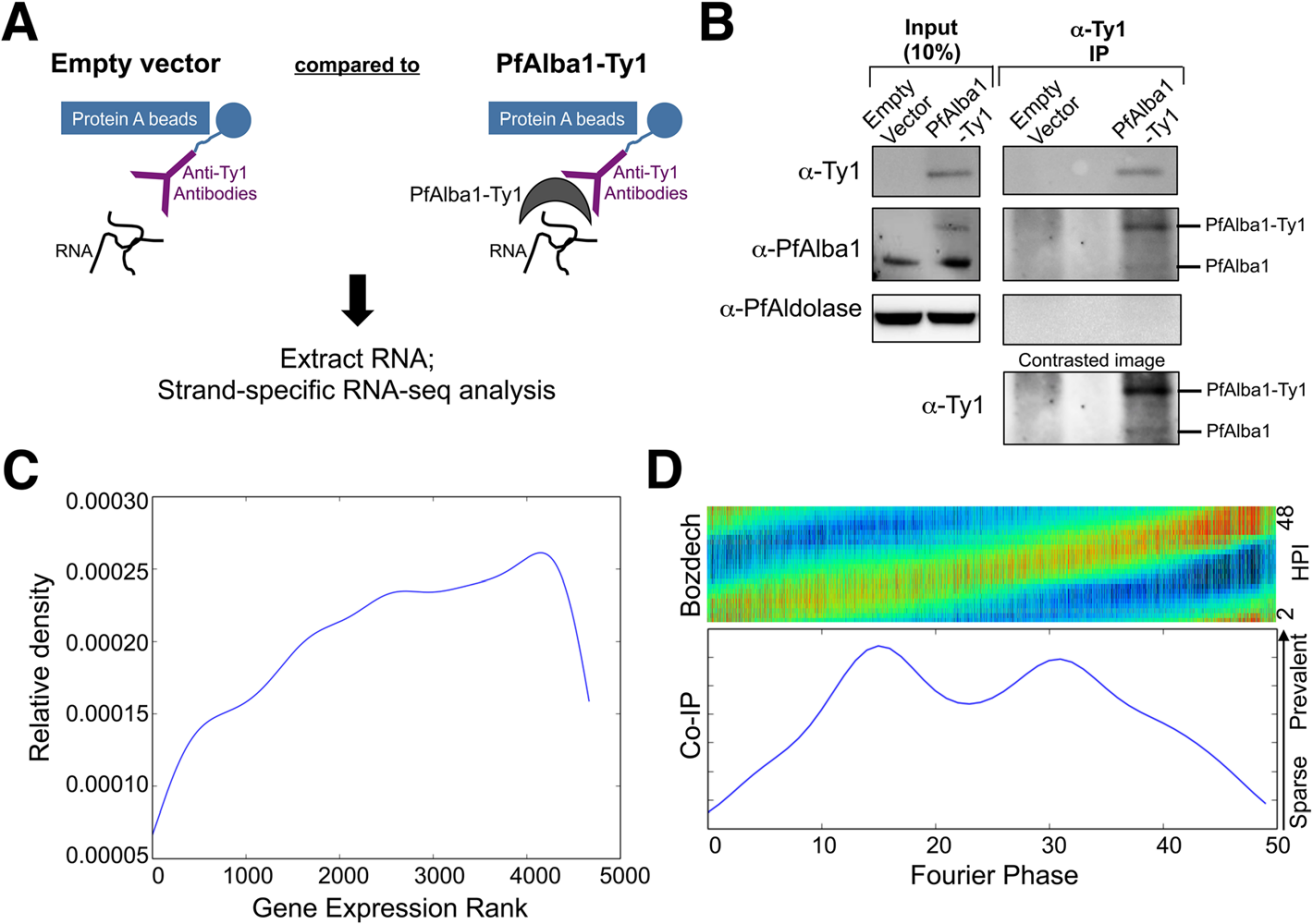
In trophozoite stages, a PfAlba1-Ty1 ribonucleoprotein complex interacts with 1665 transcripts. **a** Schematic representation of the RNA immunoprecipitation protocol. Native lysates prepared from PfAlba1-Ty1 parasites were incubated with rabbit anti-Ty1 antibodies in the presence of protease and RNAse inhibitors to immunoprecipitate (IP) a PfAlba1-Ty1-containing ribonucleoprotein complex. The IPed RNA molecules were identified by strand-specific RNA-seq. RNA IPed from empty vector parasites served as background binding. **b** Lysates (10 % of the input used for immunoprecipitation) or proteins IPed with anti-Ty1 antibodies from empty vector or PfAlba1-Ty1 transfectants were separated by denaturing gel electrophoresis and analyzed by western blotting with mouse anti-Ty1 antibodies or anti-PfAlba1 antibodies. PfAldolase served as a loading control. The contrasted image clearly shows the presence of PfAlba1 in the anti-PfAlba1-Ty1 co-IPed complex. **c** A Gaussian density kernel estimate of the distribution of gene ranks according to mRNA abundance of the 1665 transcripts that co-IPed with the PfAlba1-Ty1 complex. The *y-axis* represents relative density and the *x-axis* represents gene expression ranks, with 0 being least expressed. **d** Fourier phase distribution of the normalized RNA-seq data of the 1665 co-IPed transcripts. *Top panel*: *P. falciparum* IDC transcriptomic data were obtained from Bozdech *et* *al*. [6], where the authors provide a convenient metric, the Fourier phase, to define the hours post-infection (*HPI*) at which each gene is most abundantly transcribed. *Bottom panel*: a Gaussian density kernel estimate of the distribution of Fourier phase values of the 1665 transcripts that co-IPed with PfAlba1-Ty1 as sampled from Bozdech *et* *al*. [6]

To begin to outline PfAlba1’s role in post-transcriptional regulation, we first identified its *in vivo* RNA interactome by immunoprecipitation and deep sequencing analysis. Because attempts to immunoprecipitate (IP) endogenous PfAlba1 with anti-PfAlba1 antibodies were not successful (data not shown), we focused on PfAlba1-Ty1. A similar approach was recently used in gametocytes of the mouse malaria parasite *Plasmodium berghei* to identify the RNA targets of green fluorescent protein-tagged DOZI or CITH, the core RNA-binding components of the DOZI-CITH translational repressor complex [23]. Accordingly, we prepared cell lysates under non-denaturing conditions from trophozoite stage 3D7+PfAlba1-Ty1 parasites growing at 5 μg/ml BS — when PfAlba1 and PfAlba-Ty1 are predominantly cytoplasmic (Fig. 1c) — and IPed the tagged protein using anti-Ty1 antibodies (Fig. 3a). As shown in Fig. 3b, we were able to maximally isolate ~20 % of the total PfAlba1-Ty1 expressed in 3D7+PfAlba1-Ty1 and, to our surprise, also detected endogenous PfAlba1 in the IPed complex (see the contrasted image). Given that Alba proteins have been reported to form homo- and hetero-dimers in archaea [24, 25], one explanation for this observation may be direct interaction of PfAlba1-Ty1 with PfAlba1.

Thereafter, we performed deep sequencing of RNA (RNA-seq) in a strand-specific manner, mapped the sequencing reads to the *P. falciparum* genome, and scanned for significant peaks of binding activity using our analysis pipeline (described in detail in “Materials and methods”). Peaks that were enriched twofold or more in the PfAlba1-Ty1 immunoprecipitations compared with the empty vector immunoprecipitations were considered to represent PfAlba1-Ty1-specific binding events. We found that in trophozoite stages, 1665 transcripts co-IPed with the PfAlba1-Ty1 complex (Table S1a in Additional file 2). The co-IPed RNAs constitute 29 % of the 5672-strong parasite transcriptome (5365 coding transcripts), and include 1643 protein-coding transcripts. To weed out possible false positives and/or false negatives in our analysis, we included additional controls: we IPed protein complexes from empty vector or PfAlba1-Ty1 lysates with non-specific rabbit IgG antibodies, purified any associated RNAs, and analyzed them by strand-specific RNA-seq. When we compared the anti-Ty1 IPs from PfAlba1-Ty1 lysates with the corresponding IgG IPs using our analysis pipeline, we found that 791 transcripts were enriched in the anti-Ty1 IPs (Table S1b in Additional file 2), 612 of which (Table S1c in Additional file 2) were also present in the list of 1665 transcripts identified above. This indicated that the utilization of two different controls results in the identification of a range of possible PfAlba1 targets: a permissive list of 1665 and a conservative list of 791, with 612 high confidence targets. We decided to use the permissive list for further analysis.

We next asked if the 1665 bound transcripts were among the most highly transcribed at this stage of the IDC by performing a correlation analysis to the 28–30 h 3D7 transcriptome described by Bozdech and colleagues [6] and found that their expression ranks ranged from low to high (Fig. 3c). Furthermore, an analysis of the Fourier phase distribution of the co-IPed transcripts demonstrated that the timing of peak expression of the transcripts distributes evenly across the IDC spectrum with no obvious bias to any single hour post-infection, and closely resembles the underlying distribution described by Bozdech *etal*. [6] (Fig. 3d). Together, these data support that anti-Ty1 immunoprecipitation is not selective for the most highly transcribed trophozoite transcripts and is detecting specific PfAlba1-Ty1–mRNA complexes *in vivo*. For genes with introns, we observed that introns were spliced out in the mapped sequencing reads for the transcript, suggesting that the PfAlba1-Ty1 complex does not bind to pre-mRNA.

Lastly, Gene Ontology (GO) analysis (Figure S3a in Additional file 1; Additional file 3) and an in-depth evaluation of the co-IPed transcript list (Additional file 2) showed that housekeeping machineries, such as the ribosome and spliceosome, as well as essential cellular processes, such as glycolysis, RNA transcription, DNA replication, and protein translation, are specifically targeted by the PfAlba1-Ty1 complex at the mRNA level. This provides one explanation for our inability to genetically knockout or knockdown this gene/protein. Normalized sequencing coverage plots for some of the candidates from prominent GO classes are depicted in Figure S3b in Additional file 1.

### Recombinant PfAlba1 directly binds to the majority of transcripts that co-IP with PfAlba1-Ty1 complexes

**Fig. 4:**
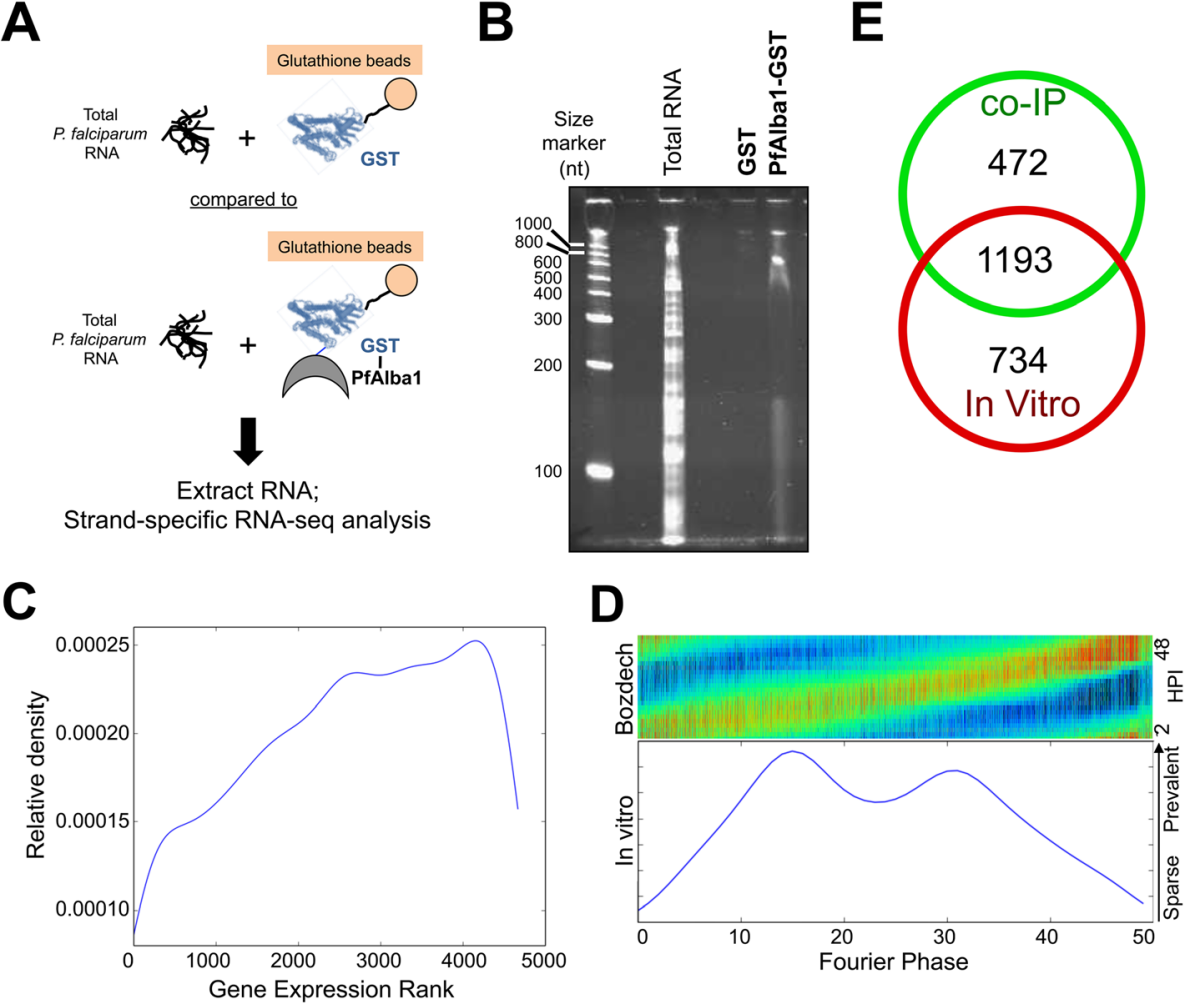
More than 71 % of the transcripts that co-IP with the PfAlba1 complex directly bind to recombinant PfAlba1 *in vitro*. **a** The *in vitro* RNA pull-down protocol. GST or GST-PfAlba1 were immobilized on glutathione-agarose magnetic beads and incubated with total *P. falciparum* RNA prepared from a mixed trophozoite and schizont culture of 3D7 in the presence of protease and RNAse inhibitors. Bound RNAs were detected by strand-specific RNA-seq, with GST serving as a negative control. **b** Total RNA, or RNAs bound to GST or GST-PfAlba1 were resolved by denaturing polyacrylamide gel electrophoresis and visualized by Sybr Gold staining. Size marker = 0.1–2 kb RNA Ladder (Invitrogen, Life Technologies; *nt =* nucleotides). **c** A Gaussian density kernel estimate of the distribution of gene ranks according to mRNA abundance of the 1993 transcripts that bound to GST-PfAlba1. Refer to legend of Fig. 3c for details. **d** Fourier phase distribution of the normalized RNA-seq data of the 1993 GST-PfAlba1-bound transcripts. Refer to legend of Fig. 3d for details. **e** Venn diagrams were used to represent the overlap of transcripts identified in the co-IPed (*co-IP*; Additional file 2) and *in* *vitro*-bound (*In vitro*; Additional file 4) datasets

To delineate transcripts that are directly bound by PfAlba1, we next adapted an *in vitro* RNA pull-down assay called specific nucleic acids associated with proteins (SNAAP) [26] with recombinant glutathione-S-transferase (GST)-PfAlba1 protein and total RNA prepared from a 3D7 trophozoite and schizont culture (Fig. 4a). As shown in Fig. 4b, the eluate from GST-PfAlba1 pull-downs was highly enriched for RNA compared with the negative control. To establish the identity of GST-PfAlba1-bound RNAs, we then performed strand-specific RNA-seq, mapped the sequencing reads to the *P. falciparum* genome, and using our analysis pipeline, discovered that 1927 RNAs were twofold or more enriched in the GST-PfAlba1 pull-downs compared with the GST control (Additional file 4). This constitutes 34 % of the parasite transcriptome and includes 1919 protein-coding transcripts. Of these, 592 and 465 transcripts reach transcription peaks in trophozoite (24–38 h) and schizont (38–48 h) stages, respectively. Moreover, the 1927 bound transcripts are not exclusively among the most highly transcribed in either of these stages (Fig. 4c) and show a broad distribution of IDC transcription peaks as determined by an analysis of their Fourier phase distribution (Fig. 4d). Together, this indicates that the binding of PfAlba1-GST to the 1927 transcripts is not dependent on their relative steady state abundance and is likely specific. Remarkably, the overlap between the *in vivo* and *in vitro* bound transcripts is 1193 or 71 % of the co-IPed transcripts (Fig. 4e), implying that a large proportion of the co-IPed transcripts is directly recognized by PfAlba1-Ty1. However, the 472 co-IPed transcripts that do not bind *in vitro* raise the possibility that PfAlba1 may associate with a few RNAs via other proteins or protein complexes.

Subsequently, we performed GO analysis to identify the cellular components, molecular functions and biological processes enriched in the *in vitro*-bound transcript dataset (Additional file 5). We found that, in addition to the classes found in the co-IPed dataset, GST-PfAlba1 directly bound to transcripts encoding components of the food vacuole, ubiquitin-dependent proteasomal catabolic process, entry into host cell, and carbohydrate biosynthetic process, to name a few (see Figure S4a in Additional file 1 for details). Normalized sequencing coverage plots of select transcripts that both co-IPed with the PfAlba1-Ty1 complex and bound *in vitro* to GST-PfAlba1 are shown in Figure S4b in Additional file 1. Overall, we conclude that, in trophozoite stages, PfAlba1 directly binds to 1193 mRNAs encoding components involved in translation, transcription, and DNA replication, and may target them for post-transcriptional regulation. Of note, translation and DNA replication are key metabolic processes that are accelerated in the trophozoite stage and need to be tightly regulated, along with hemoglobin degradation [6, 27].

### Excess PfAlba1 results in the misregulation of 926 transcripts in the trophozoite stage and the early onset of a schizont-like transcriptomic profile

**Fig. 5:**
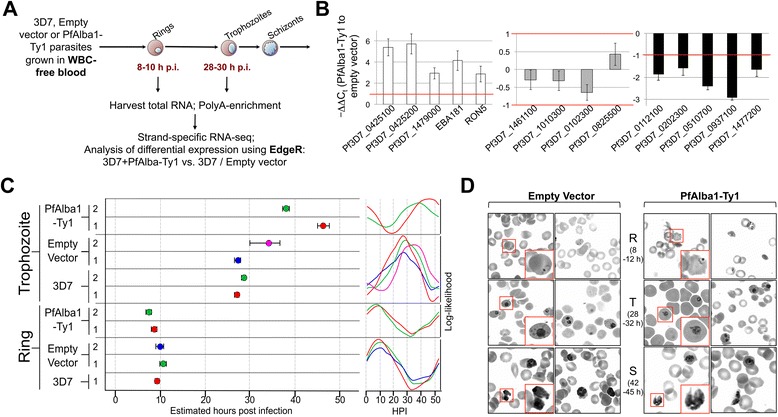
PfAlba1 overexpression perturbs the trophozoite stage transcriptome, resulting in the early onset of a schizont-like transcription profile. **a** Schematic representation of the transcriptomic profiling experiment. 3D7, 3D7+empty vector, or 3D7+PfAlba1-Ty1 parasites were grown in white blood cell (*WBC*)-free blood to ring (8–10 h post-infection (h p.i.)) or trophozoite (28–30 h p.i.) stages, and RNA harvested and mRNA enriched and analyzed by strand-specific RNA-seq. Differential expression in 3D7+PfAlba1-Ty1 relative to 3D7 and empty vector transfectants was quantified by edgeR [71]. **b** qRT-PCR analysis of the indicated genes was performed using total RNA isolated from empty vector or PfAlba1-Ty1 transfectants. The *left*, *middle* and *right panels* include genes that were upregulated, unchanged, or downregulated, respectively, according to RNA-seq. The *y-axis* denotes -ΔΔC_t_, calculated as in “Materials and methods”. Data represent the means of a minimum of three independent experiments ± standard error (*error bars*). **c** Hours post-infection (*HPI*) estimates for the transcriptomic data were obtained by passing the normalized RNA-seq data through the maximum likelihood algorithm developed by Lemieux *et* *al*. [29]. The *left panel* shows the HPI estimates within 95 % confidence intervals, while the *right panel* displays the actual likelihoods determined for each sample over the 48 h IDC. **d** Giemsa-stained blood films showing ring (*R*), trophozoite (*T*) or schizont (*S*) stages of empty vector or PfAlba1-Ty1 transfectants. Insets show zoomed-in views of the indicated infected red blood cells

It is well established that significant changes in the expression levels of an RBP can affect the stoichiometry of RBP–RNA complexes, resulting in large-scale changes in the post-transcriptional regulatory networks governed by the RBP [28]. Therefore, we postulated that measuring the steady state transcriptome of PfAlba1 overexpressing parasites would allow us to consolidate the pool of RNAs targeted by PfAlba1 and predict some of the outcomes of PfAlba1–RNA interaction. To this end, we harvested total RNA from two stages of the parasite IDC: 8–10 h rings, when PfAlba1 has a nuclear localization [20], and 28–30 h trophozoites, when PfAlba1 is predominantly cytoplasmic (Fig. 1c) [20], and performed strand-specific RNA-seq of poly(A)-enriched RNA (Fig. 5a). We found that, compared with 3D7 and 3D7+empty vector parasites, a small proportion of the ring stage transcriptome of PfAlba1-Ty1 transfectants was twofold or more differentially regulated (117 transcripts = 2.1 % of the transcriptome; 52 upregulated and 65 downregulated) with most of these transcripts (62 %) belonging to multigenic virulence gene families such as *var*, *rifin*, and *stevor* (Table S5a in Additional file 6). In contrast, 926 transcripts were twofold or more differentially regulated (635 upregulated and 291 downregulated) in 3D7+PfAlba1-Ty1 trophozoites (Table S5b in Additional file 6); these constitute 16 % of the total parasite transcriptome. To validate the transcriptional changes, we performed quantitative reverse transcriptase-PCR (qRT-PCR) on a random selection of 14 genes (Additional file 5; genes highlighted in blue) and, as shown in Fig. 5b, confirmed the changes determined by RNA-seq. More generally, these results indicate that PfAlba1 participates in maintaining the mRNA homeostasis of trophozoite stages.

We next used a maximum likelihood-based statistical analysis method [29] to estimate the developmental age of our PfAlba1-Ty1 transcriptomic datasets. Given the periodicity of gene expression in *P. falciparum* [6, 16], such an analysis separates true differential expression from cell-cycle-dependent, temporal changes in expression patterns and is an important measure of the quality of our RNA-seq replicates for technical and biological variability [29]. Notably, the 3D7+PfAlba1-Ty1 trophozoite transcriptome mapped to a later time point of the parasite lifecycle — 38–40 h or 44–46 h for the two replicates — while the timing of the ring transcriptome, at 8–10 h, was nearly identical to wild-type and empty vector-containing parasites (Fig. 5c). A multidimensional scaling plot also supported the shift in developmental age of the 3D7+PfAlba1-Ty1 trophozoite datasets (Figure S5a in Additional file 1). Taken together, these observations suggest that PfAlba1 overexpression results in the early onset of a schizont-like transcriptomic profile due to perturbation of mRNA homeostasis, and provides one explanation for the reduction of 3D7+PfAlba1-Ty1 growth. However, this does not manifest as a change in parasite DNA replication and morphology as assessed by flow cytometry (Fig. 2d) and Giemsa staining of the relevant stages (Fig. 5d), respectively.

Finally, we identified enriched GO terms in the differentially regulated dataset and they mapped to the following groups (Figure S5b in Additional file 1; Additional file 7): host cell plasma membrane, host cell cytoplasm part, microneme, rhoptry neck, inner membrane complex, heparin binding, host cell surface binding, actin binding, single organismal cell-cell adhesion, antigenic variation, and entry into host cell. A closer analysis grouped these terms into two major categories: components required for host interaction to achieve cytoadherence and components essential for erythrocyte invasion. Interestingly, only one significant GO term overlapped with the co-IPed and/or *in vitro*-bound RNA datasets: entry into host cell. In fact, we found that transcripts encoding erythrocyte invasion components including the inner membrane complex, microneme, and rhoptry proteins were all upregulated in 3D7+PfAlba1-Ty1 trophozoites. For example, 20 of ~25 rhoptry components were over threefold upregulated at this stage (Table S5b in Additional file 6; gene names are in red letters). Because these transcripts reach transcription peaks only at 38–42 h (Figure S6 in Additional file 1), we believe that the schizont-like transcriptomic shift is partly due to premature upregulation of such transcripts in PfAlba1-Ty1 trophozoites, i.e., ~8–14 h earlier than in wild-type cells.

### When PfAlba1 is overexpressed, 105 PfAlba1-bound transcripts, including 13 erythrocyte invasion components, are differentially regulated

**Fig. 6:**
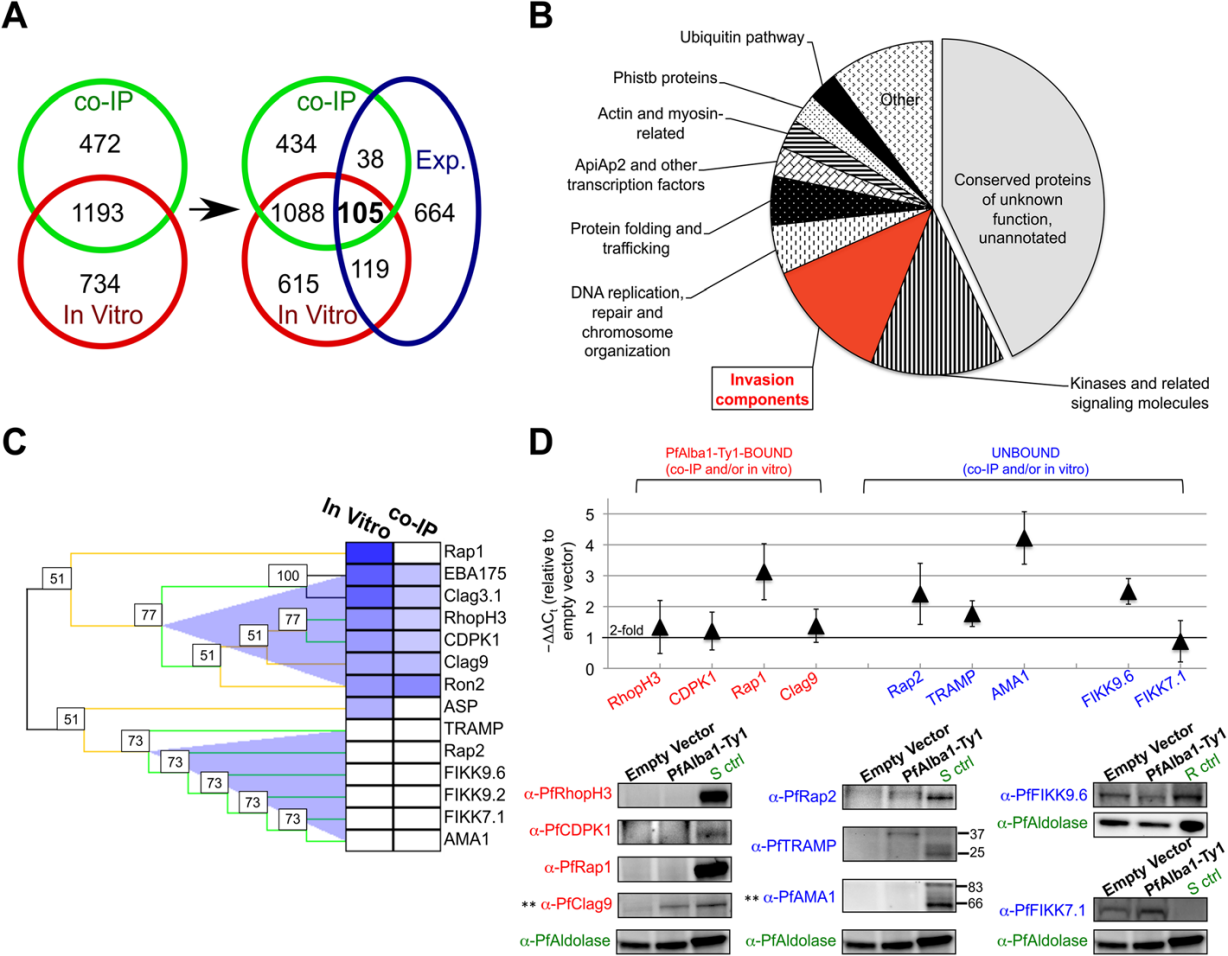
Eleven percent of the transcripts deregulated in PfAlba1-Ty1 trophozoites directly bind to PfAlba1, with binding resulting in the translational repression of select components of the erythrocyte invasion machinery. **a** Venn diagrams were used to represent the overlap of transcripts identified in the co-IPed (*co-IP*; Additional file 2), *in vitro*-bound (*In Vitro*; Additional file 4), and differentially regulated (*Exp.*; Additional file 6) datasets. **b** Pie chart showing the functionally over-represented categories in the 105 PfAlba1-bound and deregulated transcripts. **c** Bootstrap hierarchical clustering was used to partition 14 transcripts upregulated in PfAlba1-Ty1 trophozoites based on their binding (log_2_(fold change); blue shading) to PfAlba1-Ty1 (*co-IP*) and GST-PfAlba1 (*In Vitro*). The numbers in the boxes are the bootstrap scores. The resulting clusters were PfAlba1-bound (*upper cluster*) and PfAlba1-unbound (lower cluster). **d** *Top panel*: qRT-PCR analysis of the indicated genes was performed using mRNA isolated from empty vector or PfAlba1-Ty1 trophozoites. The *y-axis* denotes -ΔΔCt, calculated as in “Materials and methods”. Data represent the means of a minimum of three independent experiments ± standard error (*error bars*). *Bottom panel*: protein lysates prepared from empty vector or PfAlba1-Ty1 trophozoites were separated by denaturing gel electrophoresis and analyzed by western blotting with the indicated antibodies. Lysates prepared from either 3D7 rings (*R ctrl*) or schizonts (*S ctrl*) served as a control for protein detection, except for PfFIKK7.1. ** = Exceptions to the observed correlation between PfAlba1-RNA binding and translational repression

Having identified PfAlba1’s *in vivo* and *in vitro* RNA interactome and RNAs that are differentially regulated when parasites produce excess PfAlba1, we posited that RNAs detected in all of these datasets would likely be the first set of transcripts targeted by PfAlba1 for post-transcriptional regulation. Accordingly, we outlined their intersection using a Venn diagram and found that a small proportion, 8.8 % (n = 105), of the 1193 transcripts that bound *in vivo* and *in vitro* was twofold or more differentially regulated: 97 upregulated and 8 downregulated (Fig. 6a). Functional group analysis of the 105 transcripts revealed that 43 % encoded conserved proteins of unknown function, while the remaining encoded 13 components of the invasion machinery, such as PfCDPK1, PfEBA175, PfRhopH2, PfRhopH3, PfRON2, PfRON3, PfRON4, and PfRh2a and 2b, 14 kinases and related signaling molecules, three actin- and myosin-related proteins, and three ApiAP2 proteins, to name a few (Fig. 6b; Additional file 8). Additionally, the differentially regulated transcriptome contained 38 transcripts that only bound *in vivo* and 119 transcripts that only bound *in vitro* (Fig. 6a; Additional file 8), expanding the primary list of mRNAs targeted by PfAlba1 complexes for regulation during the IDC to 262. To visualize the cellular and functional distribution of annotated genes in the Venn intersections of Fig. 6a, we designed a schematic of a *P. falciparum* asexual blood stage cell and mapped each gene and its intersection category onto the schematic using MapMan [30] (Figure S7 in Additional file 1). It became apparent that mRNAs encoding proteins that contribute to erythrocyte invasion, including components of apical organelles and the actin- and myosin-related motor proteins, constitute a significant portion of PfAlba1 targets.

### The PfAlba1 RNA interactome contains transcripts that are described to be under translational control

In order to assign a post-transcriptional function to PfAlba1, we next compared the PfAlba1 RNA interactome with post-transcriptionally regulated gene sets identified in previous studies of asexual blood stages [16, 19]. The first dataset we evaluated was generated by Bozdech and colleagues [16] using correlative transcriptomic and proteomic analysis, and contains 127 asexual stage mRNAs that are subject to translational regulation (Table S8a in Additional file 9). We found that 104 (or 82 %) of the 127 mRNAs are bound by PfAlba1 *in vivo* and/or *in vitro* (Table S8a in Additional file 9), suggesting that PfAlba1 may modulate mRNA translation during asexual development. Thirteen of these bound RNAs are misregulated in PfAlba1-Ty1 trophozoites and include invasion transcripts such as Rap1, Rap3, RhopH2, RhopH3, and RON4. The second dataset we analyzed was generated by DeRisi and colleagues [19] by ribosomal profiling and contains 297 asexual stage mRNAs that are regulated at the level of translational efficiency (Table S8b in Additional file 9). Of these, 187 (or 63 %) are bound by PfAlba1 *in vivo* and/or *in vitro* (Table S8b in Additional file 9), again indicating a role for PfAlba1 in regulating mRNA translation. Of the bound transcripts, 48 have altered steady state levels in PfAlba1-Ty1 trophozoites and include 20 erythrocyte invasion and early ring stage transcripts such as Rap1, Rap3, RhopH2, RhopH3, Clag9, RON2-5, EBA181, MSP3, MSP6, and MSP11 (Table S8b in Additional file 9). Taken together, this analysis predicted a role for PfAlba1 in translational regulation during asexual blood stages.

### PfAlba1 binding is linked to translation repression of select erythrocyte invasion components

Next, to define the translational regulatory function of PfAlba1 during the IDC, we focused on select components of the rhoptry and invasion machinery that were upregulated in PfAlba1-Ty1 trophozoites and showed differential PfAlba1 binding in the co-immunoprecipitation and *in vitro* pull-down experiments. Namely, we focused on RhopH3, CDPK1, Rap1, and Clag9, which bound to PfAlba1 *in vivo* and/or *in vitro*, and Rap2, AMA1, and TRAMP, which did not bind in either condition (Fig. 6c). Of note, these mRNAs attain transcriptional peaks at 38–42 h [6, 16] in 3D7 parasites (Additional file 8; Figure S6 in Additional file 1) and protein expression peaks at 38–48 h [16, 17]. Additional non-invasion candidates that we analyzed and that were transcriptionally upregulated in 3D7 + PfAlba1-Ty1 parasites included members of the serine/threonine FIKK kinase family, FIKK7.1 and FIKK9.6: the mRNAs did not bind to PfAlba1 (Fig. 6c) and are maximally synthesized in mid-ring and late schizont stages, respectively (Figure S6 in Additional file 1).

We harvested 28–30 h trophozoite stage 3D7 + empty vector or 3D7 + PfAlba1-Ty1 parasites and analyzed the mRNA and protein levels of the selected genes. Using qRT-PCR analysis (Fig. 6d, upper panel), we found that the levels of all nine mRNAs were elevated in PfAlba1-Ty1 trophozoites compared with empty vector transfectants. However, using western blotting, we observed that mRNAs that bound to PfAlba1 *in vivo* and/or *in vitro*, *i.e.*, RhopH3, CDPK1, and Rap1, were not translated at this stage, while PfRap2 and PfTRAMP, whose mRNAs are not bound by PfAlba1, were translated (Fig. 6d, lower panel). The exceptions were Clag9 and AMA1, which showed the opposite trend (Fig. 6d, labeled with double asterisks) — Clag9 mRNA bound to PfAlba1, but the protein was translated in trophozoites, while AMA1 mRNA did not bind to PfAlba1, but was translationally repressed. Lastly, the FIKK proteins, whose transcripts do not bind to PfAlba1, were translated in both PfAlba1-Ty1 and empty vector trophozoites (Fig. 6d, lower panel). Overall, our results show a strong correlation between PfAlba1 association and translational repression in trophozoite stages.

### Release of translational repression between developmental stages accompanies mRNA dissociation from a PfAlba1 complex

**Fig. 7:**
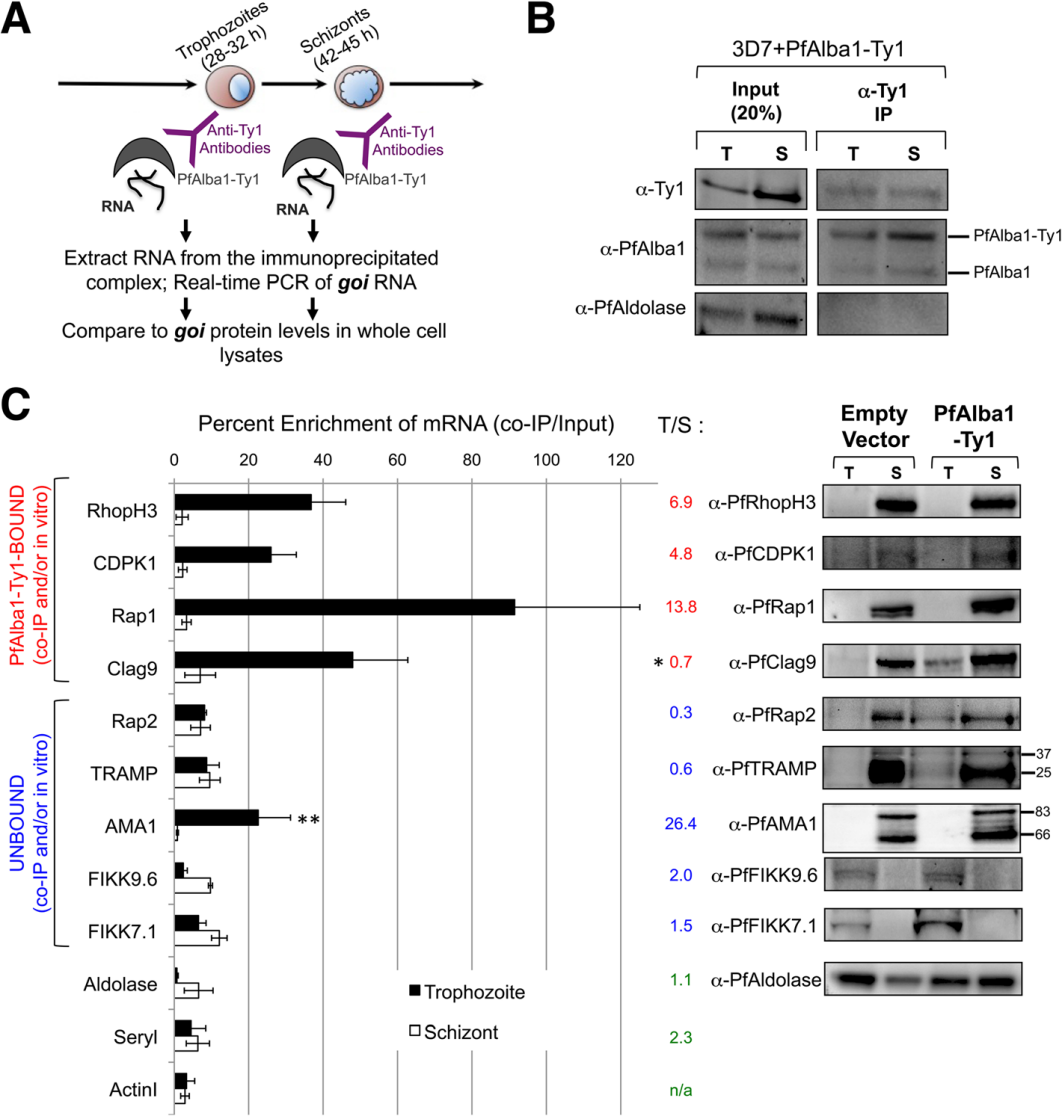
Translationally repressed transcripts associate with a PfAlba1 complex in trophozoites and are subsequently released in schizonts for efficient translation. **a** Schematic representation of the experimental setup. Native lysates prepared from PfAlba1-Ty1 parasites at the trophozoite (28–32 h) or schizont (42–45 h) stage were incubated with rabbit anti-Ty1 antibodies in the presence of protease and RNAse inhibitors to IP a PfAlba1-Ty1-containing ribonucleoprotein complex. IPed RNAs were extracted and analyzed by qRT-PCR. Finally, RNA association was compared with the presence of the corresponding protein in whole cell lysates. *goi =* gene of interest. **b** Lysates (20 % of the input used for immunoprecipitation) or proteins IPed with anti-Ty1 antibodies from PfAlba1-Ty1 trophozoite (*T*) or schizont (*S*) stages were separated by denaturing gel electrophoresis and analyzed by western blotting with mouse anti-Ty1 antibodies or anti-PfAlba1 antibodies. PfAldolase served as a control. **c** *Left panel*: IPed RNA from PfAlba1-Ty1 trophozoite or schizont lysates was analyzed by qRT-PCR with primers specific to the indicated genes. The *y-axis* of the bar graph denotes percentage enrichment, calculated as in “Materials and methods”. Data represent the means of a minimum of three independent experiments ± standard error (*error bars*). ** = The AMA1 transcript binds to PfAlba1 in trophozoite stages. Fold change of PfAlba1 association from trophozoites to schizonts, *i.e.*, T/S, was calculated as in “Materials and methods”. * = The association of PfAlba1 and Clag9 mRNA does not decrease from trophozoites to schizonts. *Right panel*: protein lysates prepared from empty vector or PfAlba1-Ty1 trophozoites (*T*) and schizonts (*S*) were separated by denaturing gel electrophoresis and analyzed by western blotting with the indicated antibodies

Finally, to investigate the molecular basis of the fine-tuning of translation of erythrocyte invasion transcripts, we assessed the association of RhopH3, CDPK1, Clag9, Rap1, Rap2, AMA1, and TRAMP mRNAs with PfAlba1-Ty1 during typical protein expression, *i.e.*, the schizont stage (42–45 h), and approximately 14–17 h before detectable protein expression, *i.e.*, trophozoites (28–30 h) (Fig. 7a). We hypothesized that invasion transcripts that are bound to and translationally repressed by PfAlba1 in trophozoites would be released from PfAlba1 in schizonts to allow for translation. We first IPed PfAlba1-Ty1 with anti-Ty1 antibodies under non-denaturing conditions (Fig. 7b), and performed qRT-PCR analysis with primers specific to the selected invasion transcripts. We included FIKK7.1 and FIKK9.6 as upregulated, but unbound, controls and analyzed three unchanged and unbound mRNAs as controls for immunoprecipitation: aldolase, actin I and seryl tRNA synthetase. For each gene, we also determined the fold change in transcript levels from trophozoite to schizont stages relative to actin I mRNA (Figure S8 in Additional file 1) and used this value to calculate the fold change in association from trophozoites to schizonts.

As illustrated in Fig. 7c (left panel), the mRNAs of RhopH3, CDPK1, and Rap1 significantly associated with PfAlba1-Ty1 in trophozoite stages, while association was lost in schizont stages. Consequently, we detected their protein products, PfRhopH3, PfCDPK1, and PfRap1, in the schizont stage alone (Fig. 7c, right panel). AMA1, which was not detected in our co-IPed/*in vitro* - bound transcript dataset (Additional files 2 and 4), significantly associated with the PfAlba1 complex in trophozoite stages alone (Fig. 7c, labeled with double asterisks), albeit to a lower extent than RhopH3, CDPK1, and Rap1, and was translationally repressed. In contrast, the mRNAs of Rap2 and TRAMP were not bound by PfAlba1-Ty1 in either trophozoites or schizonts (Fig. 7c, left panel), and the corresponding protein was detectable in both stages (Fig. 7c, right panel). The exception was Clag9: despite an apparent decrease in the association of PfAlba1-Ty1 with Clag9 mRNA from trophozoites to schizonts (Fig. 7c, left panel), the fold change of association was <1 (Fig. 7c, labeled with asterisk), and PfClag9 was translated in both stages (Fig. 7c, right panel). We surmise that PfAlba1 binding may affect other properties such as stability and/or localization of Clag9 mRNA. All control mRNAs did not significantly associate with PfAlba1-Ty1 at either stage of the IDC, and PfFIKK7.1 and PfFIKK9.6 proteins were detected only in trophozoites of both PfAlba1-Ty1 and empty vector transfectants. Taken together, we show for a number of erythrocyte invasion mRNAs that binding to and release from a PfAlba1 ribonucleoprotein (RNP) complex is associated with timely translation. This may be especially important for events that have to be programmed with high precision, such as the assembly of apical secretory organelles and erythrocyte invasion complexes in the final hours of the IDC. Indeed, we speculate that the early upregulation of the Rap1, RhopH3, AMA1, and CDPK1 transcripts observed in PfAlba1-Ty1 trophozoites is salvaged by PfAlba1’s binding to these mRNAs and preventing their translation until the late schizont stage, when the functionality of PfRap1, PfRhopH3, PfAMA1, and PfCDPK1 is crucial.

## Discussion

Gene expression during the *P. falciparum* IDC is developmentally regulated [5, 6]; however, molecular events that underlie the post-transcriptional fine-tuning of gene expression remain poorly understood. In this work, we provide experimental insight into the molecular basis of mRNA translational regulation during *P. falciparum* asexual development. Using a multipronged transcriptome-wide approach, we demonstrate that a member of the Alba DNA/RNA-binding family of proteins, PfAlba1, binds to a large portion of mRNAs in asexual blood stages and regulates mRNA homeostasis. Moreover, for a subset of genes involved in erythrocyte invasion, we demonstrate that the reversible association of PfAlba1 with mRNA follows the transition from translational repression to protein expression. Together, our observations underline a dual role for PfAlba proteins during asexual development: in addition to the previously reported perinuclear location and interaction with virulence genes in ring stages [20, 21], we establish a key contribution to RNA regulation in replicative blood stages — in particular, to modulate the timing of protein translation events required for merozoite invasion of host cells.

### PfAlba1 binds to essential blood stage RNAs either directly or via interactions with other RBPs

At the 28–30 h time point of the IDC, we find that PfAlba1 complexes co-IP 1665 transcripts, many of which encode essential cellular components involved in translation, splicing, DNA replication, and transcription, processes that are strongly associated with this stage of the IDC [6, 27]. The large repertoire of RNAs bound by PfAlba1 complexes is reminiscent of the ~1500 mRNAs that are bound by complexes containing TbRRM1, a splicing factor, in procyclic stages of the kinetoplastid parasite *Trypanosoma brucei* [31], the >9000 RNAs that are bound by the polycomb repressor complex PRC2, a chromatin regulator, in mouse embryonic stem cells [32], the mRNA interactome of poly(A)-binding proteins in yeast cells [33], and the RNA pools targeted by other eukaryotic RBPs [34]. Yet, not all RNAs bound by a given RBP complex are directly regulated by the RBP at the post-transcriptional level [34]. To identify biologically relevant interactions, immunoprecipitation studies are typically complemented with *in vitro* RNA pull-downs such as SNAAP [26], SELEX (systematic evolution of ligands by exponential enrichment) [35], and RBNS (RNA Bind-N-Seq) [36], *in vivo* assays such as PAR-CLIP (photoactivatable-ribonucleoside-enhanced crosslinking and immunoprecipitation) [37], and RBP knockdown and/or overexpression studies [34, 38]. Likewise, we performed SNAAP and found that GST-PfAlba1 directly binds to over 70 % of the co-IPed transcripts — implying that PfAlba1 is the principal RBP component of the complexes it associates with (Fig. S9a in Additional file 1, upper panel) — and used an overexpression approach to shortlist 105 primary RNA targets of PfAlba1.

Nonetheless, because 29 % of the transcripts that co-IP with PfAlba1-Ty1 are not recognized by GST-PfAlba1 *in vitro*, we speculate that other RBPs or RBP complexes may direct PfAlba1–RNA association (Figure S9a in Additional file 1, lower panel). One such complex may be the PbAlba-containing DOZI-CITH complex, which is involved in translational repression in gametocyte stages of *P. berghei* [39, 40]. A second class of RBPs that direct PfAlba1–RNA interaction may include the other *P. falciparum* Alba proteins, PfAlba2–4. This is supported by the observation that archaeal Alba proteins form homo- and hetero-dimers to bind to DNA, and putatively RNA [24, 25], while Alba proteins from other apicomplexan and kinetoplastid parasites heteromerize in an RNA-dependent manner to carry out their function [41–43]. In addition, we found that during the IDC, PfAlba4 co-IPs with PfAlba1 and 2, but not PfAlba3 (data not shown), and that PfAlba1 and PfAlba1-Ty1 co-IP (Figs. 3b and 7b). Thirdly, a member of the Bruno/CELF RBP family, PfCELP1, which localizes to multiple discrete foci in the cytoplasm of trophozoites, may associate with and regulate PfAlba1 function [44].

### PfAlba1 may act as a master regulator during *P. falciparum* asexual blood development

Considering our inability to reduce PfAlba1 levels and the large number of essential RNAs bound by PfAlba1 in trophozoite stages, it is reasonable to surmise that this RBP is a key regulator of RNA homeostasis during the *P. falciparum* IDC. Indeed, we believe that the erythrocyte invasion mRNAs characterized in this study comprise only one of many RNA regulons targeted by PfAlba1 (and maybe the other PfAlba proteins) for post-transcriptional control and that the top categories from our GO analyses (Figures. S3a, S4a, and S5a in Additional file 1) need to be further explored to assign RNA regulatory functions to PfAlba1. Moreover, because components of eukaryotic metabolic processes appear to be regulated in a coordinated manner post-transcriptionally [45], we propose that this property can be exploited to assign functionality to some of the uncharacterized mRNAs that are bound by PfAlba1. For example, some of the 43 uncharacterized mRNAs in the intersection of our various datasets in Fig. 6a may encode novel components of the invasion machinery.

Another indication of PfAlba1’s master regulatory role during the IDC is the early onset of a schizont-like transcriptomic profile upon PfAlba1 overexpression. One explanation for this may be the direct binding of PfAlba1 to the transcripts of putative DNA and/or RNA-binding proteins (Additional file 2, rows highlighted in green) and 19 ApiAP2 transcription factors (Additional file 2, rows highlighted in yellow), three of which are also upregulated in PfAlba1-Ty1 trophozoites (Additional file 8; Fig. 6b). Excess PfAlba1-mRNA binding may affect levels of the corresponding proteins and indirectly affect the trophozoite transcriptome, as is observed in PfAlba1-Ty1 trophozoites. Of note, the mRNAs of two ApiAP2 proteins implicated in transcriptional regulation of erythrocyte invasion genes, Pf3D7_0631800 [46] and Pf3D7_1007700 [47], are directly bound by PfAlba1 (Additional files 2 and 4), although the mRNA of PfBDP1, a recently identified transcriptional regulator of select erythrocyte invasion genes [46], is neither bound by PfAlba1 nor misregulated upon its overexpression.

Finally, given that PfAlba1 localizes to punctate foci in the cytoplasm of trophozoite stages, reminiscent of mRNA-containing RNP (mRNP) granules [20], and that the fate of every RNA molecule is determined by several RBPs cooperating with each other [34, 45], we speculate that PfAlba1 orchestrates RNA regulatory events by organizing its RBP partners, such as PfDOZI-CITH, PfCELF1, and PfAlba2-4, to name a few, into functional complexes. Post-translational modifications of PfAlba1 may alter the composition of PfAlba1-containing complexes in a stage-specific manner (Figure S9b in Additional file 1) and determine RNA binding specificity: phosphorylation of PfAlba1’s Alba domain has been detected in phosphoproteomic studies of schizont stages [48], whereas the effect of acetylation on DNA binding by Alba proteins is well documented in the archaea *Sulfolobus solfataricus* [49]. Ultimately, PfAlba1 may participate in complex physiological outcomes via canonical signaling pathways, such as the calcium-signaling pathway that regulates merozoite egress and invasion [50], either individually or as homo- or heteromers or in the context of mRNP granules.

### Erythrocyte invasion RNAs comprise the first set of transcripts regulated by PfAlba1 at the level of translation

Translational control is well documented in sexual and other transmission stages of malaria parasites [51]. For example, components of the *P. berghei* DOZI-CITH RNP complex were demonstrated to bind to over 730 female gametocyte mRNAs that are required for development within the mosquito vector and translationally repress a subset of them [23, 39, 40]. In addition, PfPuf2, an RBP that belongs to the Pumilio/fem3 binding factor (Puf) family was shown to bind to a UGUX_3_UA motif in the 5’ UTR and 3’ UTR, respectively, of two essential *P. falciparum* sexual stage transcripts, pfs25 and pfs28, and repress their translation [52]. However, over the past decade, apart from transcriptome- and proteome-wide studies that showed a delay in translation during the *P. falciparum* IDC [15–17] and the observation that the phosphorylation of eIF2 (eukaryotic initiation factor 2) globally represses translation in *P. falciparum* and *P. berghei* asexual blood stages [53], few molecular details have emerged about mRNA-specific regulation in asexual blood stages [54, 55]. Our results establish PfAlba1 as an mRNA-specific regulator of translational timing in asexual stages, at least for erythrocyte invasion transcripts. In fact, for four such transcripts, Rap1, RhopH3, AMA1, and CDPK1, we show that PfAlba1 binding is associated with translational repression, and postulate that eight other erythrocyte invasion transcripts found in the primary list of PfAlba1 RNA targets are similarly regulated: EBA175, RON2–4, RhopH2, Rh2a and 2b, and RALP1 (Additional file 8).

Taken together with the observation that PfAlba1’s interactome contains a majority of the translationally regulated mRNAs identified in other studies of asexual *P. falciparum* stages (see [16, 19] and the “Results” section), we propose that one of the major functions of PfAlba1 is to monitor mRNA translation, as has been described for Alba proteins from related pathogens such as *Toxoplasma gondii* [42] and *T. brucei* [43, 56]. The definitive step to address this would be from the mRNA viewpoint, *i.e.*, to determine if a given mRNA associates with different PfAlba-containing protein complexes at different points of the IDC. This is particularly important for erythrocyte invasion molecules that are being developed as malaria vaccine candidates [57].

## Conclusions

Our work on PfAlba1 provides a missing molecular link for the previously suggested delay between transcription and protein expression of a subset of genes in *P. falciparum* blood stages [17]. Indeed, our data demonstrate that biological processes such as erythrocyte invasion that require precise sequential timing of protein expression [50] depend on post-transcriptional fine-tuning of mRNA translation, in addition to being regulated at the transcriptional level [46]. Given that translation inhibitors are being developed as anti-malarials [58, 59], we speculate that specific post-transcriptional mechanisms could be potentially targeted for drug discovery. In addition, an exploration of the RNA regulons targeted by PfAlba1 may provide novel insights into parasite-specific gene regulatory mechanisms. Overall, this work highlights that the PfAlba proteins represent an important but understudied class of proteins that needs to be further explored to understand how this major human pathogen coordinates the progression of its 48-hour intra-erythrocytic development cycle.

## Materials and methods

### Parasite culture and transfection

Asexual blood stages of the *P. falciparum* laboratory strain 3D7 and its transfectants were cultured as described previously [60]. Giemsa staining of parasites to determine parasitemia and parasite developmental age was carried out as described [61].

For plasmid constructs used in this study, refer to Supplementary Materials and Methods in Additional file 1. Transfection was performed with 3D7 ring stage parasites [62] with 50 or 100 μg of the plasmid constructs. Drugs used include WR99210 (WR; Sigma) at 2.5 nM, BS (Invivogen) at 2.5 or 5 μg/ml, and TMP (Sigma) at 5 μM, unless otherwise indicated.

### Antibodies and western blotting

PfAlba1-Ty1 expression was detected using rabbit anti-Ty1 antibodies (Genscript) or mouse BB2 monoclonal anti-Ty1 antibodies [63]. Endogenous PfAlba1 expression was detected using rabbit anti-PfAlba1 antibodies [20]. For all western blot experiments, PfAldolase was used as a loading control and detected with anti-PfAldolase-HRP antibodies (AbCam). Other antibodies used in this study include: anti-PfAlba4 [20], anti-PfRap1 and anti-PfRap2 [64], anti-PfAMA1, anti-PfCDPK1, and anti-PfTRAMP (a kind gift of S. Singh and C. Chitnis, Insistut Pasteur, Paris), anti-PfRhopH3 (a kind gift of O. Puijalon, Insistut Pasteur, Paris), anti-PfFIKK9.6 [60], anti-PfFIKK7.1 (M. Nunes and A. Scherf, unpublished), and anti-PfClag9 antibodies [65]. Western blotting was performed as previously described [20]. Images were captured using a BioRad ChemiDoc system and signals quantified using ImageJ [66].

### Immunofluorescence microscopy

Asynchronous cultures of 3D7 + empty vector or 3D7 + PfAlba1-Ty1 parasites were prepared for immunofluorescence assays as previously described [20].

### Flow cytometry

To measure the growth rate of 3D7+empty vector or 3D7+PfAlba1-Ty1 parasites, a synchronous culture of ring stages was diluted to 0.2 % parasitemia in 200 μl RPMI complete medium at 4 % hematocrit. At 0 h, 24 h, 48 h, 72 h, 96 h, and 120 h, 5 μl of the culture was stained in 95 μl of D-PBS (Gibco) supplemented with 2× Sybr Green I (Ozyme; stock = 10,000×) for 30 min at room temperature, diluted 20-fold in D-PBS (final volume = 200 μl), and the Sybr Green fluorescence measured in a Guava easyCyte Flow Cytometer (EMD Millipore). We counted 10000 events in duplicate to establish an accurate parasitemia value for each culture. Data were analyzed using the InCyte software (EMD Millipore).

To analyze the 48 h replication cycle of 3D7+empty vector or 3D7+PfAlba1-Ty1 parasites, synchronous ring stages growing in RPMI complete supplemented with 5 μg/ml BS were diluted to a parasitemia of 0.5 % at 4 % hematocrit: this was considered to be the “0 h” time point. The parasitemia of the culture was subsequently analyzed every 3 h for 70 h by flow cytometry as described above. To accurately demarcate ring, trophozoite, and schizont stages, data were analyzed using FlowJo v.9 software (FlowJo, LLC).

### Immunoprecipitation of PfAlba1-Ty1-associated RNA

An equivalent of 1 × 10^9^ 3D7+empty vector or 3D7+PfAlba1-Ty1 parasites were grown to 28–30 h trophozoite stages in RPMI complete medium supplemented with 5 μg/ml BS, infected red blood cells harvested, and free parasites collected by saponin lysis. The resulting parasite pellet was lysed under non-denaturing conditions in the presence of RNAsin (Promega) and subjected to immunoprecipitation analysis with rabbit anti-Ty1 antibodies or rabbit IgG antibodies (Sigma). IPed RNA was directly used to prepare strand-specific RNA-seq libraries (see below), without poly(A) enrichment. A minimum of two biological replicates was sequenced for each experimental and control immunoprecipitation. See Supplementary Materials and Methods in Additional file 1 for more details.

### Isolation of RNAs that bind to PfAlba1-GST *in vitro*

To identify RNAs that bind to PfAlba1 *in vitro*, the SNAAP method proposed by Trifillis *et al.* [26] was followed. Modifications included the use of recombinant GST or PfAlba1-GST [20] as starting material, and the analysis of bound RNA by strand-specific RNA-seq (see below), without poly(A) enrichment. A minimum of two biological replicates was sequenced for each experimental and control sample. To visualize the bound RNA on a gel, the RNAs were size-separated by denaturing gel electrophoresis in a 6 % TBE-Urea gel (Life Technologies) and visualized by staining with SybrGold (Life Technologies).

### Preparation of strand-specific RNA-seq libraries and sequencing

Strand-specific Illumina sequencing libraries were prepared as described [11] using 14–20 cycles of library amplification. The resulting preparations were sequenced using a 75 or 100 nucleotide single-end run on a HiSeq 2000 (Illumina).

For transcriptomic analysis, total RNA from synchronized 3D7, 3D7+empty vector, or 3D7+PfAlba1-Ty1 parasites at the ring (8–10 h) and trophozoite (28–30 h) stages was prepared using the miRNeasy mini kit (Qiagen) according to the manufacturer’s instructions; to reduce human RNA contamination, the parasites were grown in white blood cell-free blood in RPMI complete medium supplemented with 5 μg/ml BS. Next, ~10–15 μg total RNA was treated with DNase using the Turbo DNA-free kit (Life Technologies), poly(A)-enriched using the Dynabeads mRNA purification kit (Life Technologies), and used for strand-specific RNA-seq library preparation. A minimum of two biological replicates was analyzed for each experimental and control sample.

### Analysis of RNA-seq data

Quality control of fastq files was performed using the FastQC software [67]. Sequencing reads were mapped to the *P. falciparum* 3D7 genome (v.3, GeneDB) using the BWA MEM algorithm under default settings [68]. Properties of the datasets generated in this study and related statistics are provided in Additional file 10.

Samples obtained from the immunoprecipitation and *in vitro* binding experiments were treated differently from the transcriptomic data (see below). Here, the goal was to identify genes of interest (GOIs) that were enriched in PfAlba1-bound samples, relative to the control. Each sample was submitted to the following pipeline (Figure S10 in Additional file 1): enriched peaks were identified using MACS2 software [69] after controlling for a false discovery rate of 5 % (default setting of MACS2) using Benjamini and Hochberg’s correction method [70]; peak data were normalized to reads per million; a peak-to-GOI network was constructed, with edges being created only if a peak overlapped a GOI; the log_2_(fold change) was calculated for each peak for each contrasting sample pair (co-IPed or *in vitro*-bound versus corresponding control); GOIs were given an aggregate fold-change score by taking the mean of associated peaks; GOIs with twofold or more associated peaks in the bound sample were further analyzed. Thus, only genes containing significant peaks of protein binding with signal at least 100 % above background noise were considered for further analysis.

After initial normalization and processing with edgeR [71] (Figure S10 in Additional file 1), the transcriptomic data were explored using the plotMDS function from the R:limma modeling package [72], which showed consistent clustering of replicates and meaningful separation of clusters by time and genotype (Figure S5a in Additional file 1). The resulting multidimensional scaling plot represents each sample in two dimensions, with the distance between two samples reflecting log_2_(fold change) in expression. The edgeR pipeline was then followed to compare PfAlba1-Ty1 datasets (n = 2) to control samples (n = 3 to 4) and to select transcripts that were twofold or more differentially regulated genes in either ring or trophozoite stages at a significance level of ≥95 %, after correcting for a false discovery rate of 5 %. A total of 117 and 926 GOIs were thus selected (Additional file 6) in the ring and trophozoite datasets, respectively.

GO analysis of the enriched GOIs in each experiment was performed using GeneDB GO definitions and the BiNGO plugin [73] for Cytoscape [74].

### qRT-PCR analysis

To determine the levels of different transcripts in 3D7+empty vector or 3D7+PfAlba1-Ty1 parasites growing in RPMI complete medium supplemented with 5 μg/ml BS, ~300 μl of infected red blood cells at a parasitemia of 2–5 % 28–30 h trophozoites were harvested and total RNA prepared using the miRNeasy mini kit. Next, qRT-PCR analysis was performed according to Zhang *et al*. [75]. Seryl-tRNA synthetase (seryl; PF3D7_0717700) and PfActin I (ActI; PF3D7_1246200) were used as internal controls. The normalized transcript level of a GOI was calculated using the equation: ΔCt = Ct_goi_ – Ct_seryl (or) actI_. The fold change of a transcript between 3D7+empty vector and 3D7+PfAlba1-Ty1 parasites was calculated as: ΔΔC_t_ = ΔCt(of PfAlba1-Ty1) – ΔCt(of empty vector). All qRT-PCR primers used in this study are listed in Additional file 11.

To compare the RNAs associated with PfAlba1-Ty1 in trophozoite (28–30 h) and schizont (42–45 h) stages, PfAlba1-Ty1–RNA complexes were IPed from synchronized parasites as previously described, and bound RNA extracted using the miRNeasy kit and purified in a final volume of 25 μl. Simultaneously, 50 μl of the cleared lysate was used to purify “input” RNA (corresponding to 10 % of the total RNA used for immunoprecipitation) in a final volume of 25 μl. qRT-PCR analysis of the IPed and input RNA was performed as in Zhang *etal*. [75]. The percentage enrichment of an IPed transcript was calculated using the equation: E = 100 × 2^(adjusted input − Ct_IP_), where adjusted input is Ct_Input_ – 3.332, to account for a dilution factor of 10. The fold change in association of a transcript between trophozoites (T) and schizonts (S) was calculated as: Fold change = E_T_/(E_S_ × 2^−(ΔCt(of Schizont input) − ΔCt(of Trophozoite input))), where ΔCt = Ct_goi_ – Ct_actI_.

### Availability of supporting data

The datasets, *i.e.*, fastq files, supporting the results of this article are available in the EMBL-EBI European Nucleotide Archive [ENA:PRJEB9611; secondary accession number ERP010738] [76].

## Additional files

Additional file 1: **Supplementary Materials and Methods.** **Figure S1**Gene knockout of *ALBA1* and protein knockdown of PfAlba1 using the eDHFR destabilization domain were not successful. **Figure S2** Increase in the levels of PfAlba1-Ty1 and endogenous PfAlba1 in response to increasing BS concentration results in growth defects of 3D7 + PfAlba1-Ty1 parasites. **Figure S3** Analysis of the transcripts that co-IPed with a PfAlba1-Ty1 complex in PfAlba1-Ty1 trophozoites. **Figure S4** Analysis of the transcripts that bound to recombinant GST-PfAlba1 *in vitro*. **Figure S5** Analysis of the transcripts that were differentially regulated in PfAlba1-Ty1 trophozoites. **Figure S6** Plotting the gene classes present in the intersections of the co-IP, *in vitro*-bound, and differentially regulated datasets using MapMan. **Figure S7** Transcription profiles for select mRNAs that were probed for their translation status in PfAlba1-Ty1 trophozoites. **Figure S8** Levels of several invasion transcripts increases in PfAlba1-Ty1 schizonts relative to trophozoites. **Figure S9** Proposed model for PfAlba1 function. **Figure S10** RNA-seq data analysis pipeline. (DOCX 5465 kb)

Additional file 2: Table S1. List of transcripts that co-immunoprecipitated with PfAlba1-Ty1 complexes in trophozoite stages. (XLS 435 kb)

Additional file 3: Table S2. Gene Ontology (GO) analysis of transcripts that co-immunoprecipitated with PfAlba1-Ty1 complexes in trophozoites. (XLS 192 kb)

Additional file 4: Table S3. List of transcripts that bound to GST-PfAlba1 *in vitro*. (XLS 232 kb)

Additional file 5: Table S4. Gene Ontology (GO) analysis of transcripts that bound to GST-PfAlba1 *in vitro*. (XLS 266 kb)

Additional file 6: Table S5. Fold-change in the steady state level of every *Plasmodium falciparum* transcript in 3D7+PfAlba1-Ty1 ring or trophozoite stages relative to 3D7 and 3D7+empty vector parasites. (XLS 841 kb)

Additional file 7: Table S6. Gene Ontology (GO) analysis of 926 transcripts that were differentially regulated in PfAlba1-Ty1 trophozoites. (XLS 115 kb)

Additional file 8: Table S7. List of transcripts that were over twofold differentially regulated in PfAlba1-Ty1 trophozoites and that were bound directly by PfAlba1 *in vivo* and *in vitro*. (XLS 42 kb)

Additional file 9: Table S8. List of transcripts identified by Foth *et al*. [16] and Caro *et al*. [19] as being translationally regulated in *Plasmodium falciparum* asexual blood stages. (XLS 80 kb)

Additional file 10: Table S9. Properties of fastq files generated using RNA-seq and relevant statistics. (XLS 51 kb)

Additional file 11: Table S10. List of primers used in the study. (XLS 33 kb)

## Abbreviations

BS: blasticidin-S
GO: Gene Ontology
GOI: gene of interest
GST: glutathione-S-transferase
IDC: intra-erythrocytic development cycle
IP: immunoprecipitate
mRNP: mRNA-containing ribonucleoprotein
qRT-PCR: quantitative reverse transcriptase-polymerase chain reaction
RBP: RNA-binding protein
RGG: arginine/glycine-rich
RNP: ribonucleoprotein
SNAAP: specific nucleic acids associated with proteins
TMP: trimethoprim
UTR: untranslated region
