# Supplementary material for: The PfAlba1 RNA-binding protein is an important regulator of translational timing in Plasmodium falciparum blood stages
Source: Genome Biol. 2015 Sep 28;16:212. doi: 10.1186/s13059-015-0771-5 (PMC4587749; doi:10.1186/s13059-015-0771-5)
Supplement: Supplementary file 1 — Supplementary Materials and Methods. Figure S1Gene knockout of ALBA1 and protein knockdown of PfAlba1 using the eDHFR destabilization domain were not successful. Figure S2 Increase in the levels of PfAlba1-Ty1 and endogenous PfAlba1 in response to increasing BS concentration results in growth defects of 3D7 + PfAlba1-Ty1 parasites. Figure S3 Analysis of the transcripts that co-IPed with a PfAlba1-Ty1 complex in PfAlba1-Ty1 trophozoites. Figure S4 Analysis of the transcripts that bound to recombinant GST-PfAlba1 in vitro. Figure S5 Analysis of the transcripts that were differentially regulated in PfAlba1-Ty1 trophozoites. Figure S6 Plotting the gene classes present in the intersections of the co-IP, in vitro-bound, and differentially regulated datasets using MapMan. Figure S7 Transcription profiles for select mRNAs that were probed for their translation status in PfAlba1-Ty1 trophozoites. Figure S8 Levels of several invasion transcripts increases in PfAlba1-Ty1 schizonts relative to trophozoites. Figure S9 Proposed model for PfAlba1 function. Figure S10 RNA-seq data analysis pipeline. (DOCX 5465 kb) [file 13059_2015_771_MOESM1_ESM.docx]

**Supplementary Information for Vembar *et al.***

**Supplementary Materials and Methods**

***Generation of plasmid constructs***

Genomic DNA was prepared from a mixed stage culture of 3D7 using the DNeasy Blood and Tissue kit (Qiagen) according to manufacturer’s instructions. Gene knockout constructs for *ALBA1* (*PF08_0074* or *PF3D7_0814200*) were created by inserting sequences with homology to the *ALBA1* coding sequence (cds), or the 5’ and 3’ UTRs of the *ALBA1* cds, at either end of the human dihydrofolate reductase (hDHFR) gene in the pCC1 vector [1]. Following transfection of 3D7 parasites, the plasmids were selected with WR99210 (WR).

The protein knockdown construct for PfAlba1 was generated as follows. First, the pGDB integration vector described by Goldberg and colleagues [2] was modified to: 1) contain the hDHFR cassette, and 2) carry a deletion of the GFP coding sequence upstream of the *Escherichia* *coli* DHFR destabilization domain (eDHFR; Fig. S1A). The resultant pGDB_2_WR vector could now be selected for with three different drugs: WR, Blasticidin-S (BS) and trimethoprim (TMP). Next, a part of the *ALBA1* cds was amplified from 3D7 genomic DNA using primers 074-pGDB_2_WR-Fwd and 074-pGDB_2_WR-Rev (Table S1) and inserted upstream of the eDHFR cassette in pGDB_2_WR, generating pGDB_2_WR-Alba1. Both the empty vector and pGDB_2_WR-Alba1 were independently transfected into 3D7 parasites and selected with BS at 5 μg/ml. Transfectant parasites appeared after 15-20 days of drug selection. When the cultures reached a parasitemia of 1%, they were split in two: one fraction was maintained with BS alone, while the other fraction was treated with BS and TMP to promote integration. Once integrant *ALBA1-eDHFR-HA* parasites were obtained (Fig. S1B), the culture was grown with TMP, but without BS, to get rid of any non-integrated plasmid, and *ALBA1-eDHFR-HA* clones were selected by dilution cloning.

The pLN-Ty1C  “empty vector” was constructed by replacing the pfenr-GFP sequence contained in the pLN-ENR-GFP plasmid [3] with a nucleotide sequence encoding for two repeats of the Ty1 epitope (LEVHTNQDPLD) followed by a STOP codon. The pPfAlba1-Ty1C construct, which was previously reported [4], was generated by inserting the coding region of *ALBA1* without the STOP codon, upstream of the Ty1 epitope in pLN-Ty1C. Both the empty vector and pPfAlba1-Ty1C were independently transfected into 3D7 parasites and selected with BS at 2.5 μg/ml. Transfectant 3D7+empty vector or 3D7+PfAlba1-Ty1 parasites appeared after 20-25 days of drug pressure.

- ***Immunoprecipitation of PfAlba1-Ty1-associated RNA***
- An equivalent of 1*10^9^ 3D7+empty vector or 3D7+PfAlba1-Ty1 parasites were grown to 28-30 h trophozoite stages in RPMI complete medium supplemented with 5 μg/ml BS, infected RBCs harvested, and free parasites collected by saponin lysis. The resulting parasite pellet was resuspended in 1.5 ml of IP/lysis buffer (50 mM Tris pH 7.4, 150 mM NaCl, 1 mM EDTA, 1 mM EGTA, 1% Triton X-100) freshly supplemented with protease inhibitors and RNAsin (Promega), and incubated at 4°C for 1 h with rotation. After lysis, unbroken cells and cell debris were pelleted at 13000 rpm at 4°C for 15 min. 500 μl of the cleared lysate was immediately mixed with 2.5 μg of rabbit anti-Ty1 antibodies and 2.5 μl of RNAsin and incubated at 4°C overnight with rotation. The next day, precipitates were removed by centrifugation at 13000 rpm at 4°C for 15 min and the cleared supernatant mixed with 40 μl of a 50% slurry of protein A-sepharose (PAS) beads (GE Healthcare) in IP/lysis buffer. The mixture was incubated at 4°C for 2 h with rotation and PAS beads collected by centrifugation at 2000 rpm at 4°C for 2 min. The beads were washed twice with ice-cold IP/lysis buffer and twice with ice-cold wash buffer (10 mM Tris-HCl, pH 7.5, 1.5 mM MgCl_2_, 150 mM KCl, 0.1% Triton X-100). Finally, immunoprecipitated (IPed) RNA was eluted with 350 μl of Qiazol provided with the miRNeasy mini kit (Qiagen) and purified in a volume of 21.5 μl according to manufacturer’s instructions. This was directly used to prepare strand-specific RNA-seq libraries [5], without polyA enrichment. A minimum of two biological replicates was sequenced for each experimental and control sample from the co-immunoprecipitation experiment. To analyze IPed protein, the washed beads were directly resuspended in 25 μl of 2X XT sample buffer and the eluate analyzed by western blotting.
-
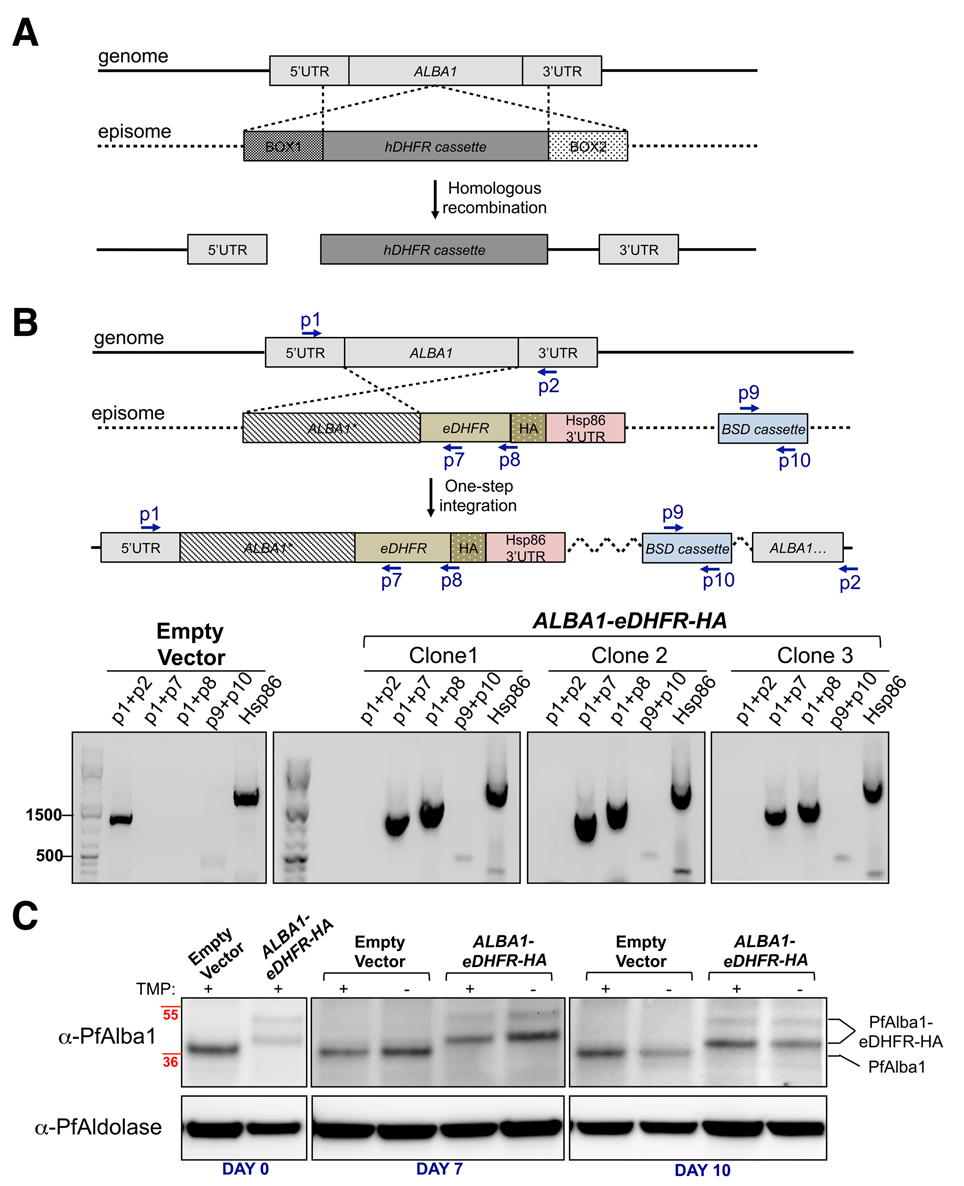
**Supplemental Figures**
- **Figure S1**: **Gene knockout of *ALBA1* and protein knockdown of PfAlba1 using the eDHFR destabilization domain were not successful. A.** Schematic representation of the gene knockout strategy. The aim was to replace the *ALBA1* genomic locus with the human dihydrofolate reductase (DHFR) selection cassette by homologous recombination. **B.** Top panel: In the protein knockdown approach, *ALBA1* was replaced with *ALBA1-eDHFR-HA* and the 3’UTR of the Hsp86 gene by single crossover plasmid integration. The resulting protein contained the *Escherichia coli* eDHFR domain, which is stabilized by the small molecule Trimethoprim (TMP). Bottom panel: The integration of the pGDB_2_WR-Alba1 plasmid at the *ALBA1* locus was confirmed by PCR of genomic DNA prepared from clones of the resultant ALBA1-eDHFR-HA parasite line. Primer pairs targeted endogenous *ALBA1* (p1+p2; See Table S1 for primer sequences), the transgenic *ALBA1-eDHFR-HA* locus (p1+p7 and p1+p8), the blasticidin deaminase (*BSD*) selection cassette (p9+p10) and the unrelated *HSP86* genomic locus. 3D7 parasites harboring the empty vector pGDB_2_WR served as a negative control. The scheme (top panel) is not drawn to scale. M = GeneRuler 1 kb Plus DNA Ladder (Fermentas, Life Technologies) **C.** Empty vector or ALBA1-eDHFR-HA parasites were grown in the presence (+) or absence (-) of the stabilizing drug TMP for the indicated number of days (0, 7, 10) and harvested. Subsequently, protein lysates were separated by denaturing gel electrophoresis and PfAlba1 or PfAlba1-eDHFR-HA detected by western blotting with anti-PfAlba1 antibodies. PfAldolase served as a loading control.
-
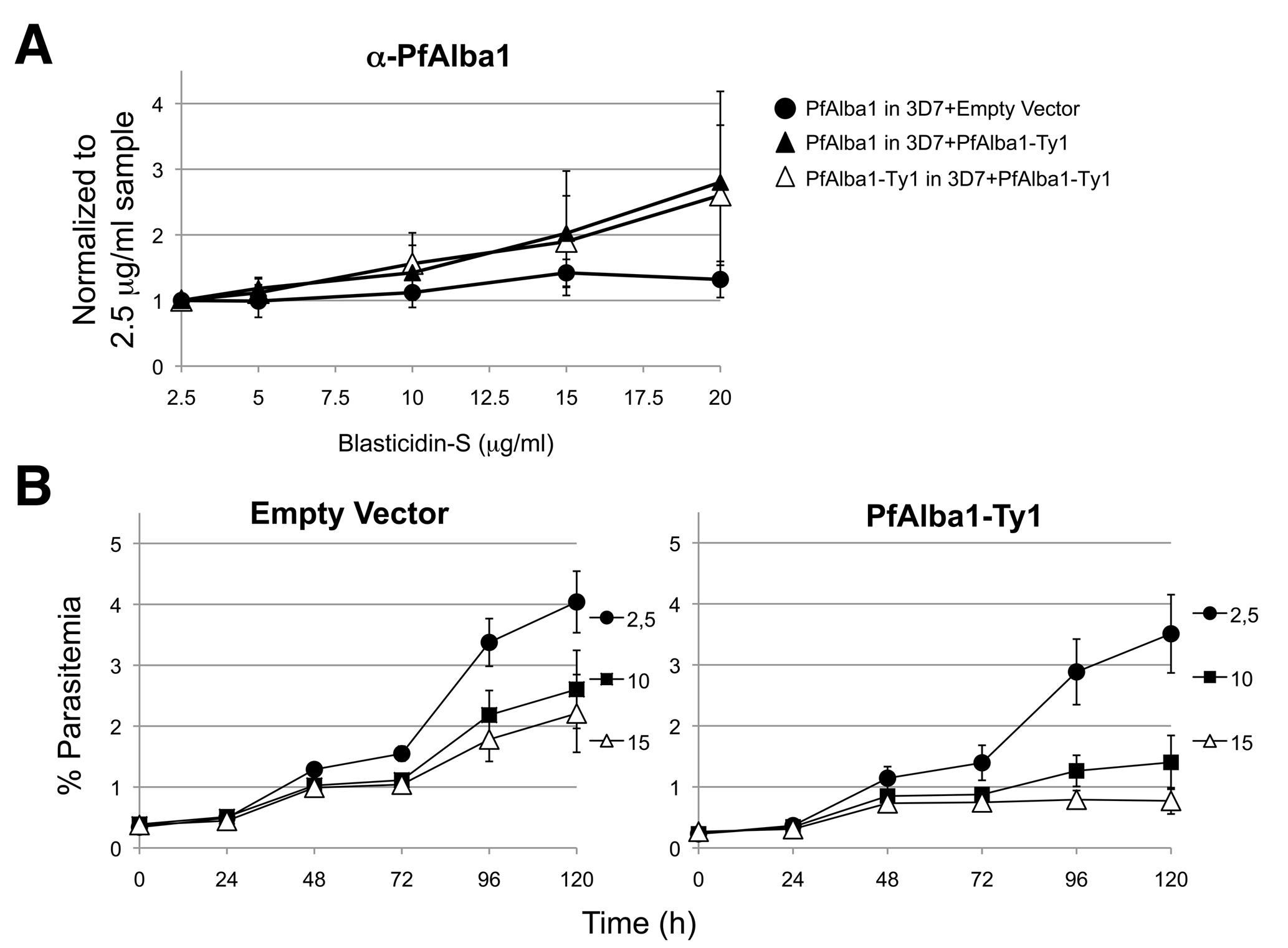


**Figure S2**: **Increase in the levels of PfAlba1-Ty1 and endogenous PfAlba1 in response to increasing BS concentration results in growth defects of 3D7+PfAlba1-Ty1 parasites. A.** The levels of PfAlba1-Ty1 and PfAlba1, as detected by the anti-PfAlba1 antibodies in Fig. 2A in empty vector (3D7+pLN-Ty1) and PfAlba1-Ty1 (3D7+PfAlba1-Ty1) transfectants, were measured by densitometry. For each BS concentration, after normalizing the PfAlba1 signal to the PfAldolase signal, data were normalized to the 2.5 μg/ml sample. Data represent the means of a minimum of three independent experiments ± S.E. (error bars). **B.** The growth of empty vector (left panel) or PfAlba1-Ty1 (right panel) parasites was measured by flow cytometry for 5 days in the presence of the indicated concentrations of BS (μg/ml). The y-axis denotes the percentage parasitemia at each time point. Data represent the means of a minimum of three independent experiments ± S.E. (error bars).

-
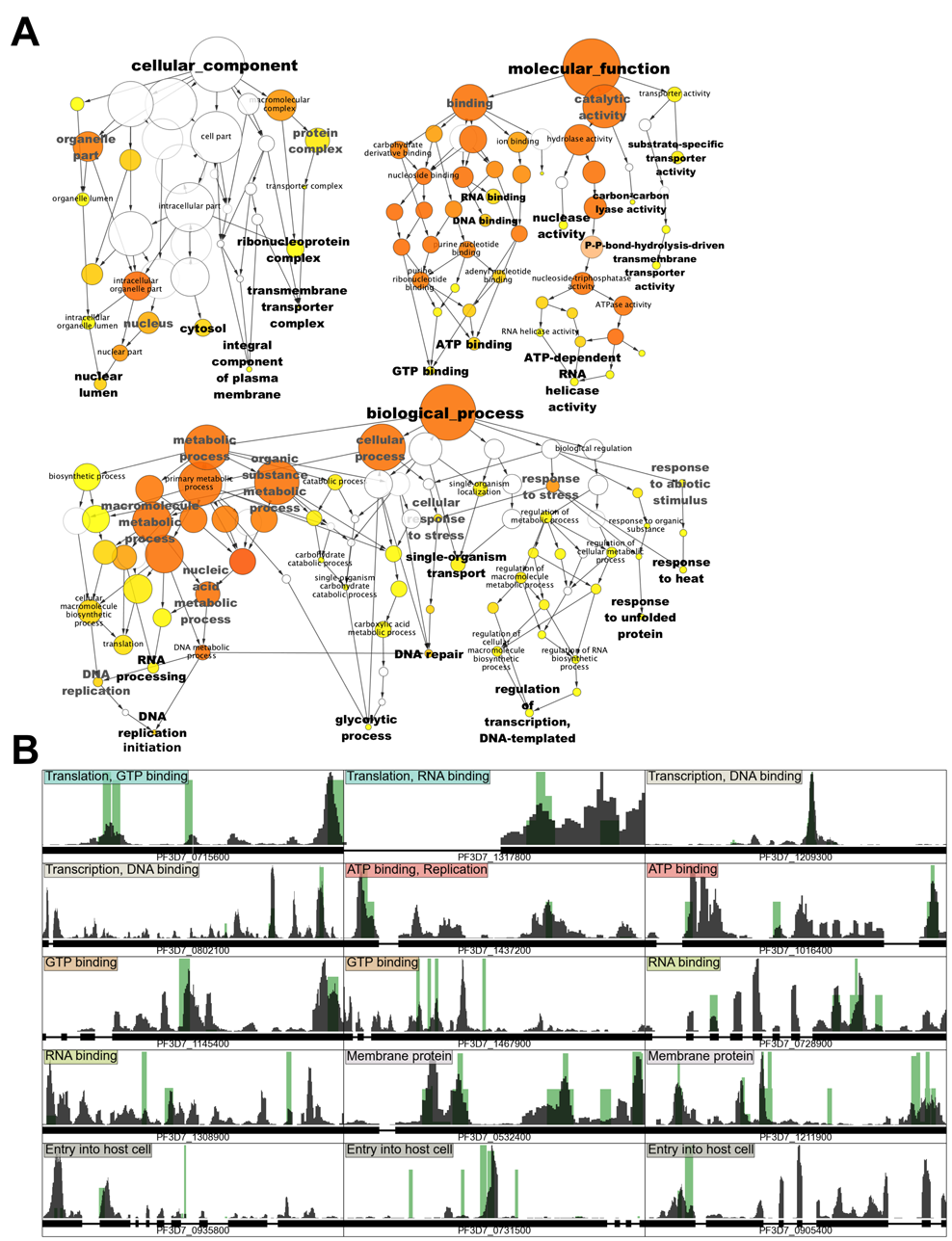


**Figure S3**: **Analysis of the transcripts that co-IPed with a PfAlba1-Ty1 complex in PfAlba1-Ty1 trophozoites.** **A.** Gene ontology (GO) term analysis of the co-IPed transcripts in three GO categories: cellular component, molecular function and biological process. Parts of the GO network that contain over-represented terms are shown, with the size of a GO term node indicative of the number of transcripts that are associated with it. The color of a node is indicative of its significance: where white is not significant (p>0.05), and yellow to orange ranges from least to most significant. **B.** Representative coverage plots from the normalized RNA-seq data for 15 genes from different GO classes. The GO term(s) associated with each gene is indicated. For each gene, the x-axis represents the gene body with exon-intron structure shown while the y-axis represents the normalized reads scaled between 0 and 1. Empty vector = green bars; PfAlba1-Ty1 = dark gray bars.

-
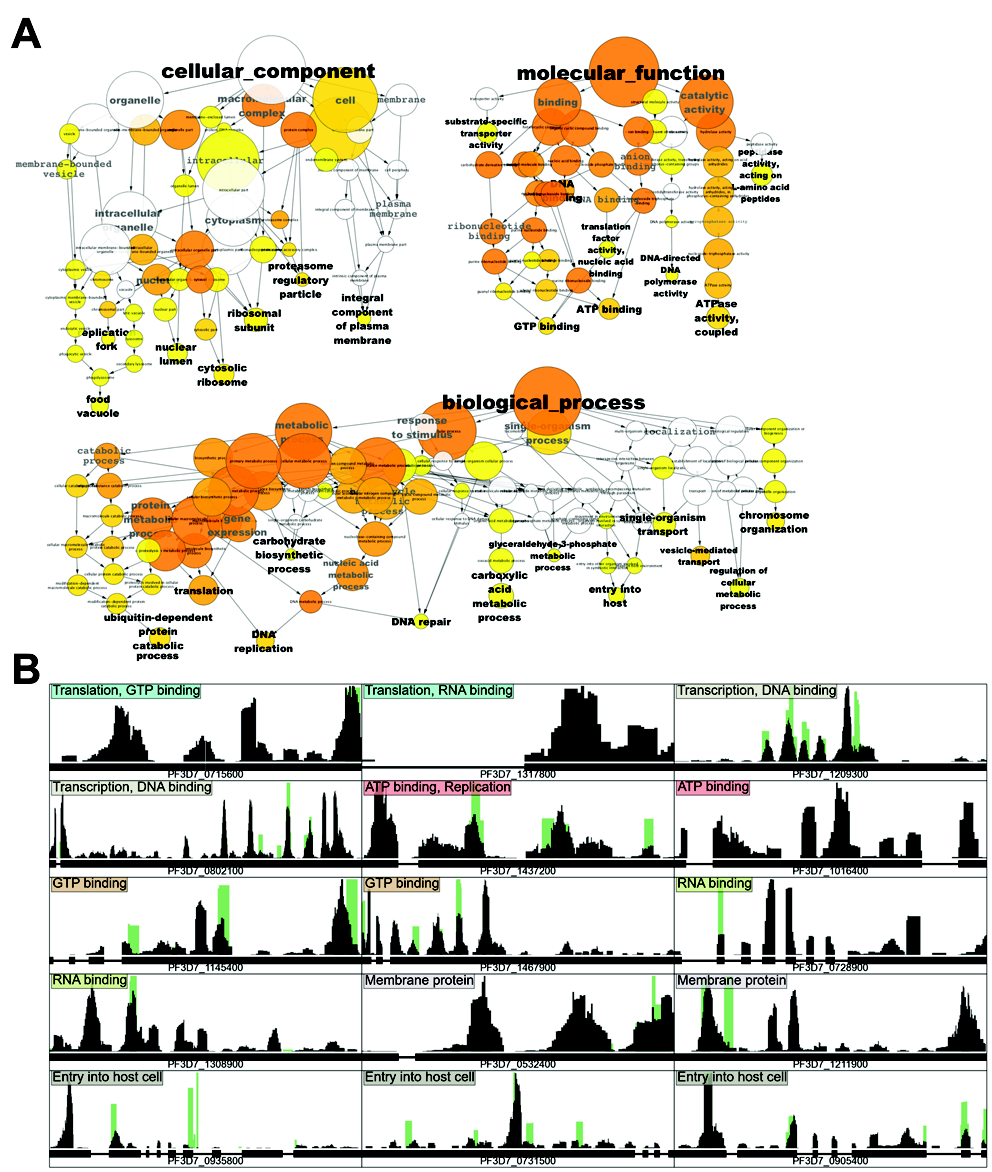

- **Figure S4**: **Analysis of the transcripts that bound to recombinant GST-PfAlba1 *in vitro*.** **A.** Gene ontology (GO) term analysis of the *in vitro*-bound transcripts in three GO categories: cellular component, molecular function and biological process. Parts of the GO network that contain over-represented terms are shown, with the size of a GO term node indicative of the number of transcripts that are associated with it. The color of a node is indicative of its significance: where white is not significant (p>0.05), and yellow to orange ranges from least to most significant. **B.** Representative coverage plots from the normalized RNA-seq data for the 15 genes that co-IPed with PfAlba1-Ty1 in Fig. S3B. The GO term(s) associated with each gene is indicated. For each gene, the x-axis represents the gene body with exon-intron structure shown while the y-axis represents the normalized reads scaled between 0 and 1. Empty vector = green bars; PfAlba1-Ty1 = dark gray bars.
-
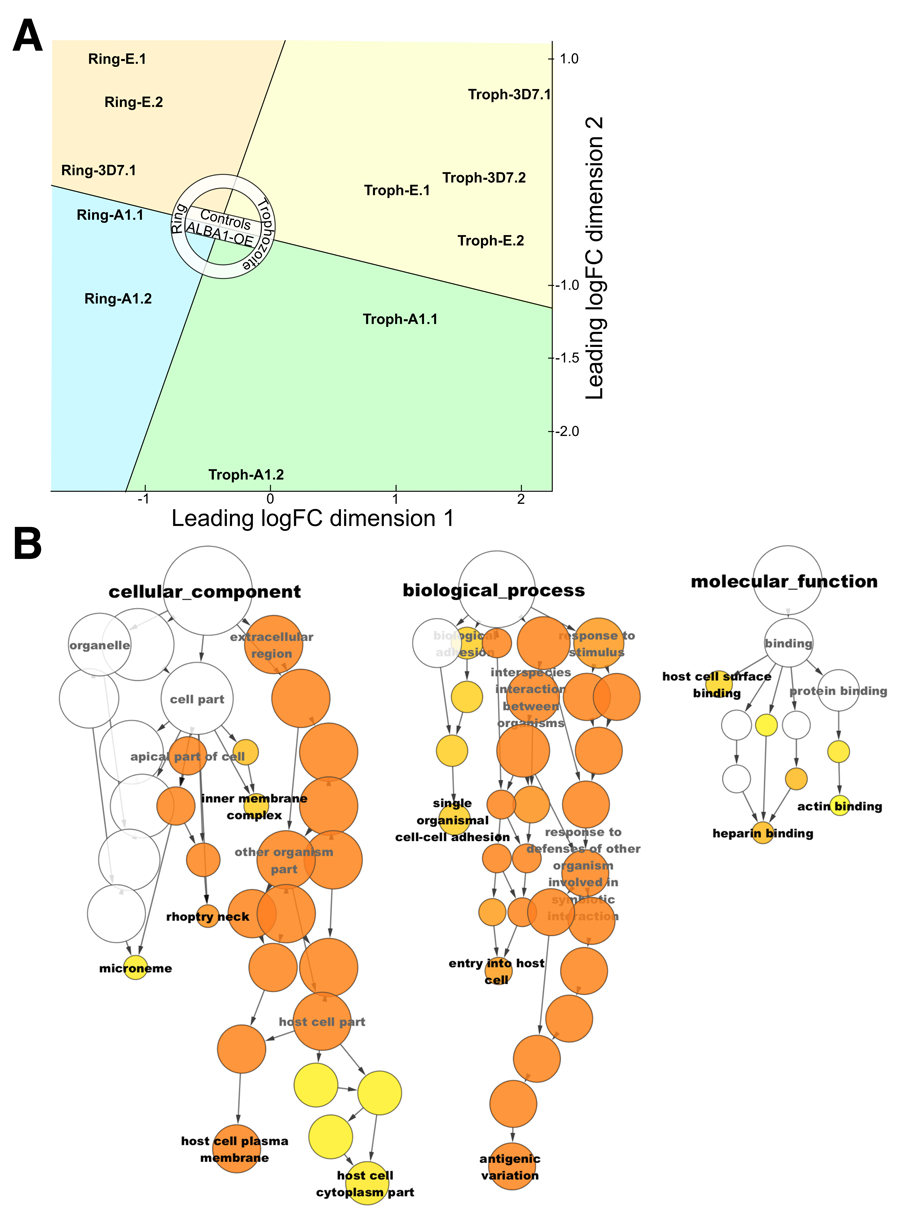

- **Figure S5:** **Analysis of the transcripts that were differentially regulated in PfAlba1-Ty1 trophozoites. A.** A multidimensional scaling plot representing each RNA-seq dataset in two dimensions, such that the distances between each dataset reflect the typical log_2_(fold change) (logFC) between them. Areas of the plot have been demarcated to highlight the clustering of similar datasets and emphasize the separation of samples into natural groups, by time and/or genotype. Ring-3D7.1 = 3D7 rings; Ring-E.1 and Ring-E.2 = 3D7+empty vector rings; Ring-A1.2 and Ring-A1.3 = 3D7+PfAlba1-Ty1 rings; Troph-3D7.1 and Troph-3D7.2 = 3D7 trophozoites; Troph-E.1 and Troph-E.2 = 3D7+empty vector trophozoites; Troph-A1.1 and Troph-A1.2 = 3D7+PfAlba1-Ty1 trophozoites. **B.** Gene ontology (GO) term analysis of the differentially regulated transcripts in three GO categories: cellular component, molecular function and biological process. Parts of the GO network that contain over-represented terms are shown, with the size of a GO term node indicative of the number of transcripts that are associated with it. The color of a node is indicative of its significance: where white is not significant (p>0.05), and yellow to orange ranges from least to most significant.


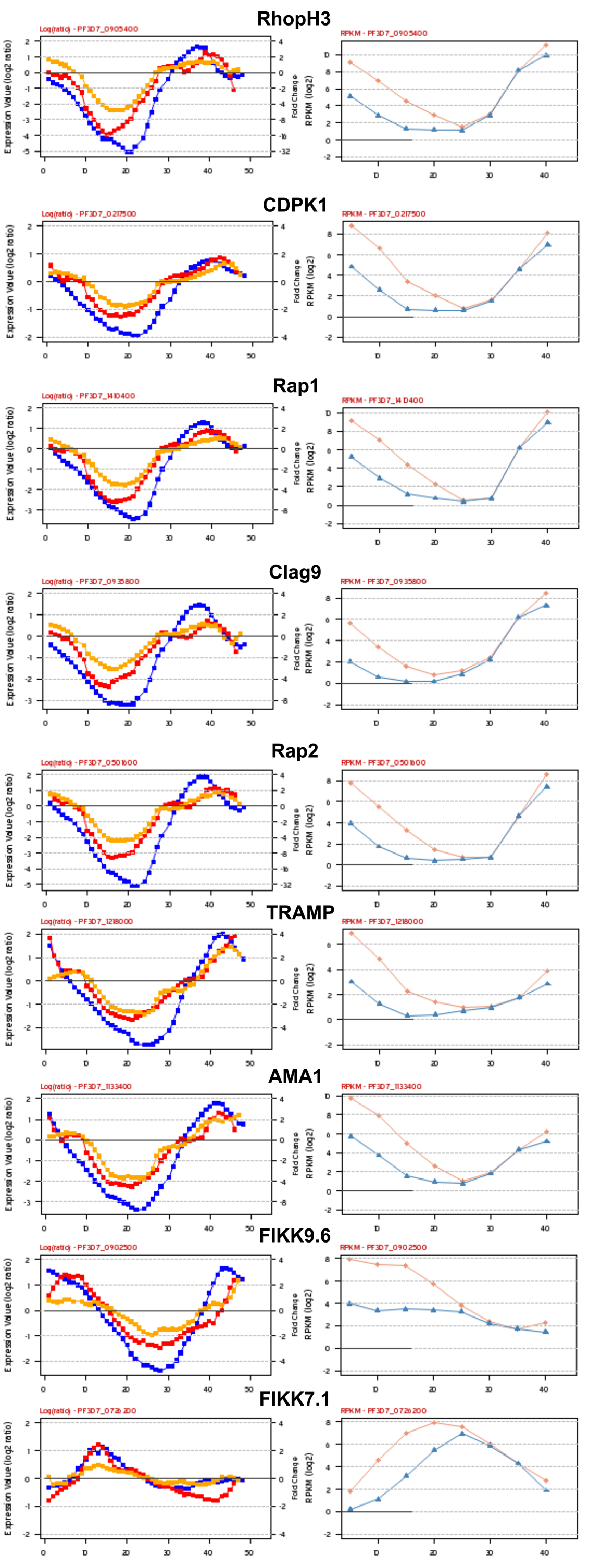


- **Figure S6: Transcription profiles for select mRNAs that were probed for their translation status in PfAlba1-Ty1 trophozoites.** The transcription profiles of the indicated genes were extracted from plasmodb.org. Left panel = Microarray analysis of the *P. falciparum* IDC [7]. Right panel = RNA-seq analysis of the IDC [8]. The y-axis represents the expression of each transcript, while the x-axis represents time (in h).


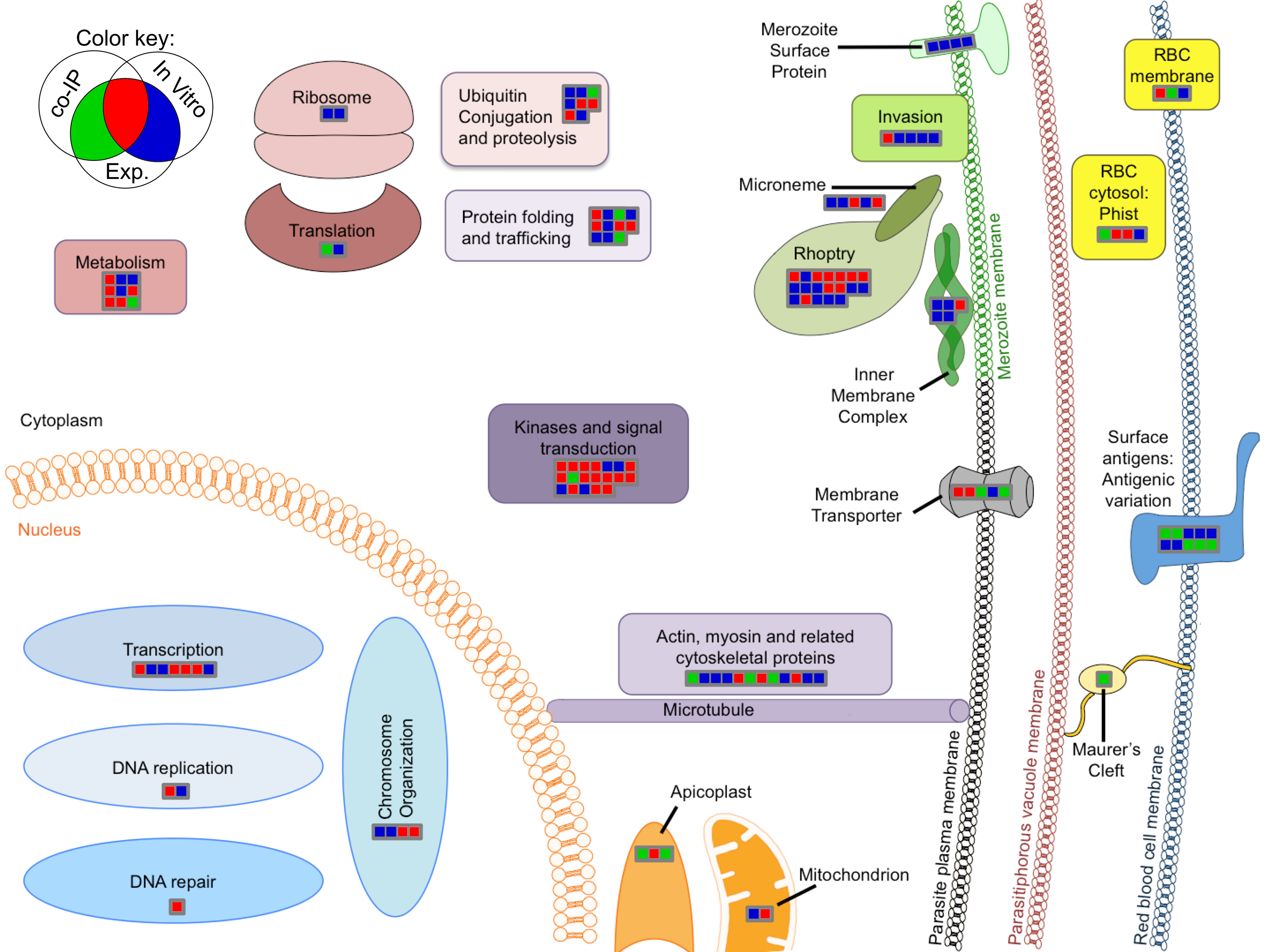


- **Figure S7: Plotting the gene classes present in the intersections of the co-IP, *in vitro*-bound and differentially regulated datasets using MapMan.** A drawing of a *P. falciparum* cell was used to illustrate the different gene classes that may be targeted by PfAlba1 for regulation. Each gene is represented as a square. A Venn diagram in the top left corner of the figure describes the color-coding system used to depict the intersection category of each gene. An interactive version of this figure can be visualized using the MapMan software [6] (http://mapman.gabipd.org/web/guest/mapman) with the files and instructions provided in the Vembar.MapManPackage.zip folder.
-
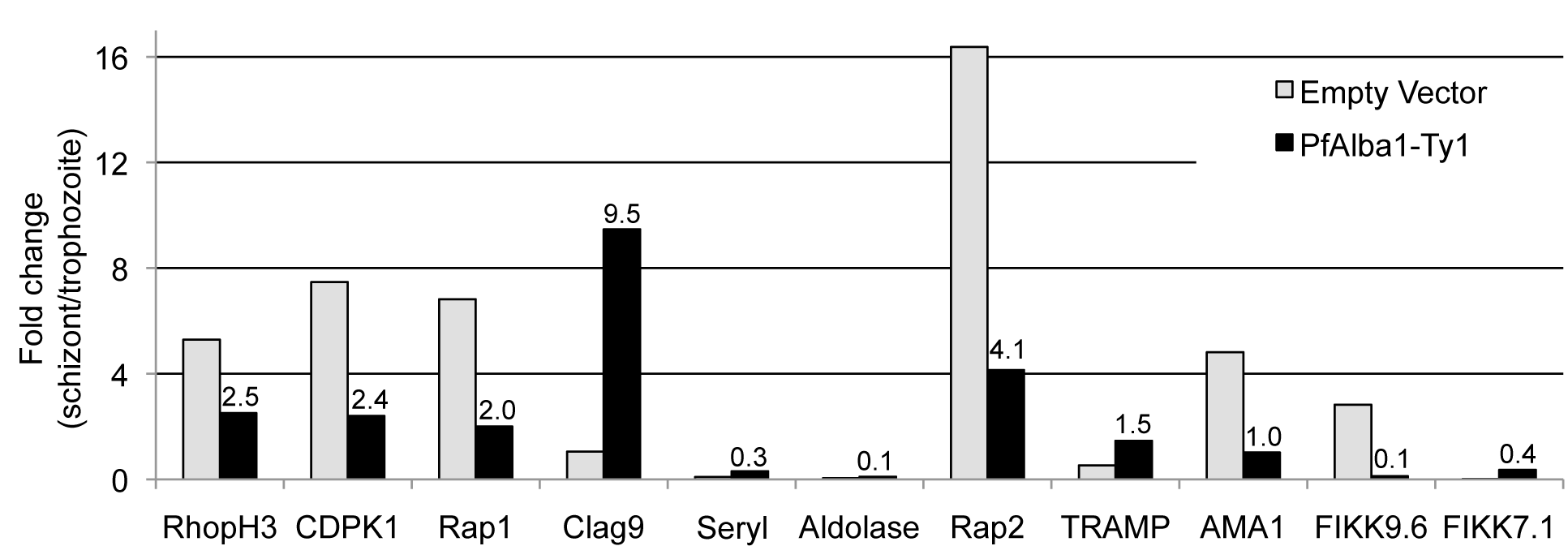

- **Figure S8: Levels of several invasion transcripts increases in PfAlba1-Ty1 schizonts relative to trophozoites.** “Input” RNA was purified from native lysates of trophozoites (28-32 h) or schizonts (42-45 h) of empty vector (gray columns) or PfAlba1-Ty1 transfectants (black columns) and mRNA levels of the indicated genes analyzed by qRT-PCR. The y-axis represents the fold change in transcript levels in schizonts relative to trophozoites and was calculated using the formula: 2^(ΔCt(Schizont input) – ΔCt(Trophozoite input)) as described in the main text. The actual value of fold change for PfAlba1-Ty1 transfectants is indicated. Data represent the means of a minimum of two independent experiments.
-
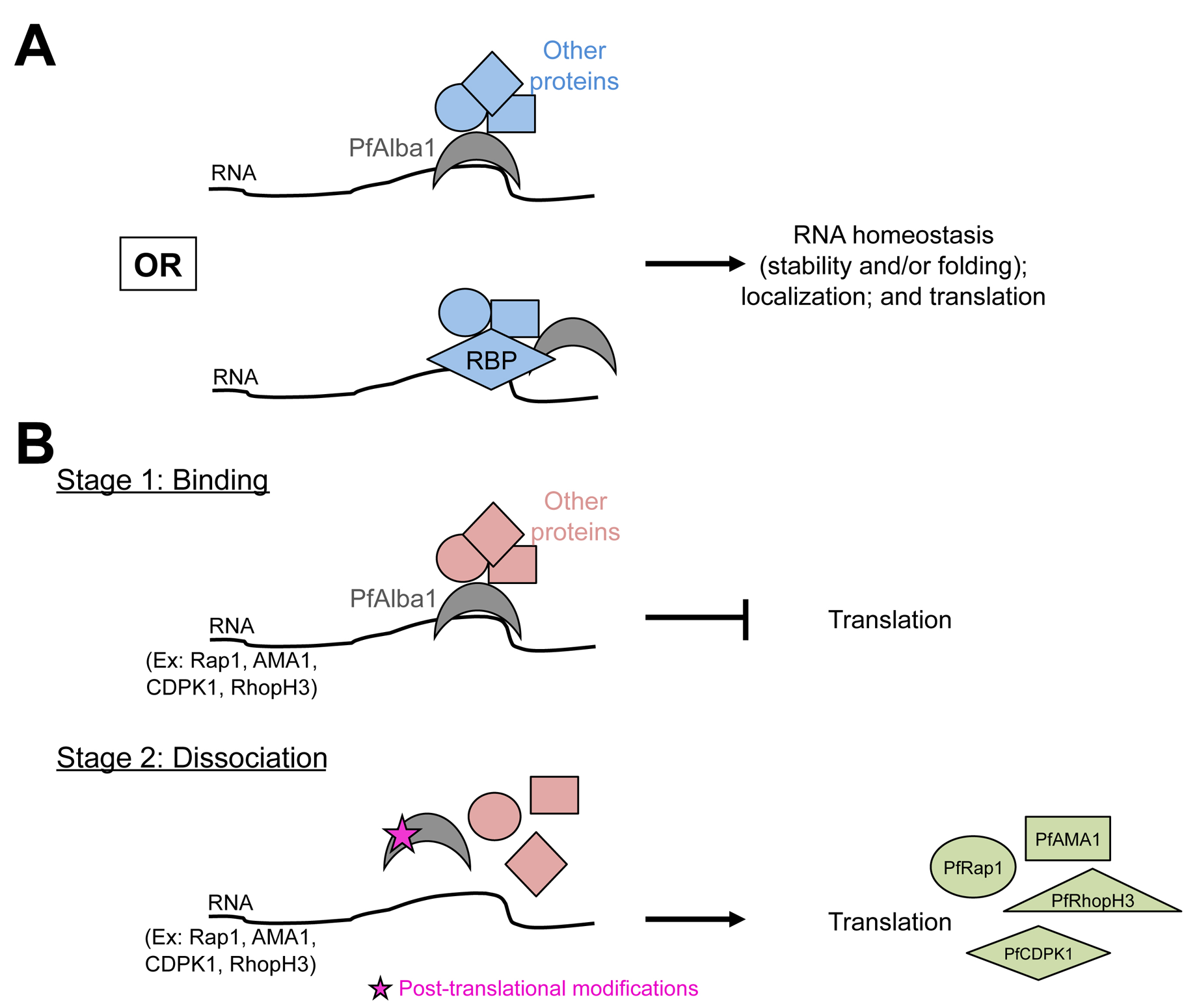

- **Figure S9: Proposed model for PfAlba1 function.** Our data suggest that PfAlba1 plays a role in, **A.** RNA homeostasis of trophozoite stages, and, **B.** Translational repression of select mRNAs encoding erythrocyte invasion components.


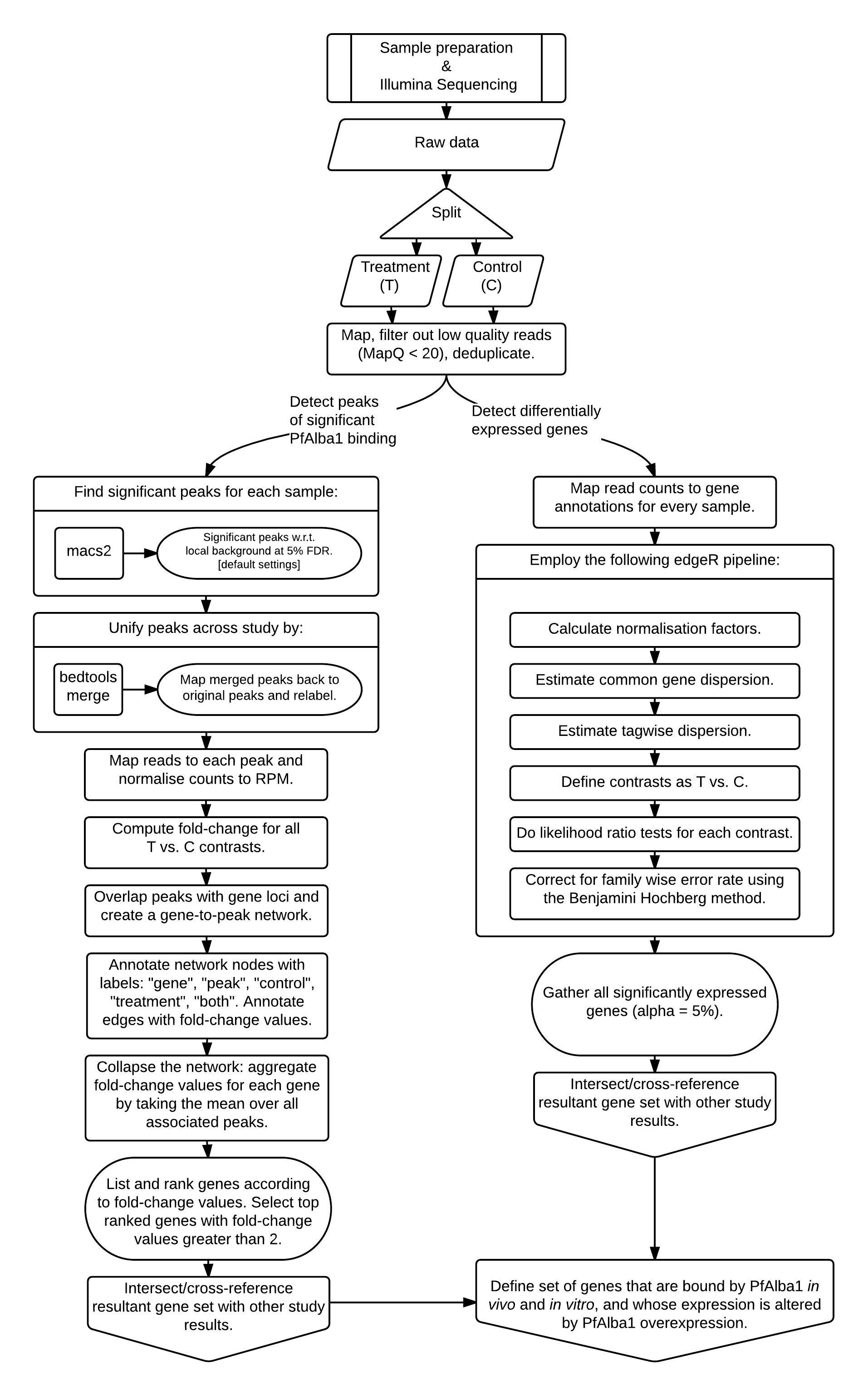


**Figure S10: RNA-seq data analysis pipeline.** A flowchart describing the data analysis pipeline used to identify transcripts significantly bound by PfAlba1 in the co-IP and *in vitro* binding assays (left panel) and transcripts differentially regulated upon PfAlba1 overexpression (right panel). See Material and Methods for additional details.

**List of References:**

1. Maier A.G., Rug M., O’Neill M.T., Brown M., Chakravorty S., Szestak T., Chesson J., Wu Y., Hughes K., Coppel R.L. et al. (2008). Exported proteins required for virulence and rigidity of Plasmodium falciparum-infected human erythrocytes. Cell. *134*, 48-61.

2. Muralidharan V., Oksman A., Iwamoto M., Wandless T.J., and Goldberg D.E. (2011). Asparagine repeat function in a Plasmodium falciparum protein assessed via a regulatable fluorescent affinity tag. Proc Natl Acad Sci U S A. *108*, 4411-4416.

3. Nkrumah L.J., Muhle R.A., Moura P.A., Ghosh P., Hatfull G.F., Jacobs W.R.J., and Fidock D.A. (2006). Efficient site-specific integration in Plasmodium falciparum chromosomes mediated by mycobacteriophage Bxb1 integrase. Nat Methods. *3*, 615-621.

4. Chene A., Vembar S.S., Riviere L., Lopez-Rubio J.J., Claes A., Siegel T.N., Sakamoto H., Scheidig-Benatar C., Hernandez-Rivas R., and Scherf A. (2012). PfAlbas constitute a new eukaryotic DNA/RNA-binding protein family in malaria parasites. Nucleic Acids Res. *40*, 3066-3077.

5. Siegel T.N., Hon C.C., Zhang Q., Lopez-Rubio J.J., Scheidig-Benatar C., Martins R.M., Sismeiro O., Coppee J.Y., and Scherf A. (2014). Strand-specific RNA-Seq reveals widespread and developmentally regulated transcription of natural antisense transcripts in Plasmodium falciparum. BMC Genomics. *15*, 150.

6. Thimm O., Blasing O., Gibon Y., Nagel A., Meyer S., Kruger P., Selbig J., Muller L.A., Rhee S.Y., and Stitt M. (2004). MAPMAN: a user-driven tool to display genomics data sets onto diagrams of metabolic pathways and other biological processes. Plant J. *37*, 914-939.

7. Bozdech Z., Llinas M., Pulliam B.L., Wong E.D., Zhu J., and DeRisi J.L. (2003). The transcriptome of the intraerythrocytic developmental cycle of Plasmodium falciparum. PLoS Biol. *1*, E5.

8. Otto T.D., Wilinski D., Assefa S., Keane T.M., Sarry L.R., Bohme U., Lemieux J., Barrell B., Pain A., Berriman M. et al. (2010). New insights into the blood-stage transcriptome of Plasmodium falciparum using RNA-Seq. Mol Microbiol. *76*, 12-24.
